# Use of fermented spent coffee grounds as a substrate supplement for rearing black soldier fly larvae, *Hermetia illucens* (L), (Diptera: Stratiomyidae)

**DOI:** 10.7717/peerj.14340

**Published:** 2022-10-31

**Authors:** Kanyanat Khaekratoke, Parichart Laksanawimol, Anchana Thancharoen

**Affiliations:** 1 Department of Entomology, Kasetsart University, Bangkok, Thailand; 2 Faculty of Science, Chandrakasem Rajabhat University, Bangkok, Thailand

**Keywords:** SCG, Bioconversion rate, Fermented substrate, Growth performances

## Abstract

**Background:**

Spent coffee grounds (SCG), an increasingly abundant waste product with environmental disposal problems, has been used as a dietary supplement for many animals and have the potential to be used as a dietary supplement for black soldier fly (BSF) larvae; however, its effective use is still under scrutiny. To date, no studies have considered the use of SCG after microbial fermentation (fSCG) and its effects on BSF life history.

**Methods:**

A mixture of fruit and vegetable pulp residue supplemented with one of six different fSCG percentages (0%, 20%, 40%, 60%, 80%, and 100% by weight) were provided as a diet substrate in order to evaluate the effect of the fSCG quantity on BSF growth, yield, and conversion ability.

**Results:**

The addition of fSCG to the pulp diet prolonged larval development times, while 100% fSCG affected the larval survival rate and resulted in a male-biased adult sex ratio. The 20–40% fSCG and 40–60% fSCG treatments supported the largest prepupal and mature larval sizes, respectively. The highest waste reduction efficiency and feed conversion rate by BSF larvae was found with 20% fSCG, similar to the control (0% fSCG).

**Discussion:**

From the short rearing time, high yield, and high bioconversion efficiency, a 20% fSCG supplementation of the mixed pulp was recommended for rearing BSF larvae. These data are valuable for coffee by-product waste management in urban areas.

## Introduction

Globally, coffee consumption has increased, while the production of instant coffee has doubled in recent years, which has resulted in an overwhelming increase in spent coffee grounds (SCG) waste (Stylianou et al., 2018). Although the valuable utilization of SCG has recently expanded, including in bioethanol, biodiesel, and biogas production (Atabani et al., 2019; Blinová, Bartošová & Sirotiak, 2017; Caetano et al., 2017; Kwon, Yi & Jeon, 2013), 46% of all SCG waste currently is still disposed of in landfills, causing environmental pollution due to its toxic phenolic compounds. However, SCG has a high organic matter content, especially carbohydrates (45.3%), in the form of hemicellulose (36.7%) and cellulose (8.6%) (Mussatto et al., 2011), and protein (8.5–13.6%) (Belitz & Grosch, 1999). In addition, SCG can be processed to reduce its toxicity using multiple methods (*e.g*., warm treatment, microbial biodegradation, and aerobic fermentation), and has then been evaluated for alternative uses. Thus, suitable processing of SCG could be developed to allow alternative uses to reduce waste and environmental pollution.

The black soldier fly (BSF), *Hermetia illucens* (L.) (Diptera: Stratiomyidae), has recently attracted attention as its larval stages bioconvert high amounts of food waste into larval biomass that can then be utilized as a high-quality, alternative protein source for food and feeds (Amrul et al., 2022; Barragán-Fonseca, Dicke & van Loon, 2017). The BSF larvae (BSFL) composition has a high ratio of protein (35–49%) and fat (5–50%) (Addeo et al., 2021; Fuso et al., 2021). Recently, BSFL have been evaluated for use as an animal feed for poultry, pigs, aquaculture, and even dogs (Barragán-Fonseca, Dicke & van Loon, 2017; Bosch & Swanson, 2020).

The BSFL can ingest various kinds of organic wastes, such as food waste, industrial by-products, abattoir waste, millet flakes, manure, and human faeces including sewage sludge (Chia et al., 2020; Chia et al., 2018; Dobermann, Field & Michaelson, 2019; Ewusie et al., 2018; Lalander et al., 2019). Some difficult-to-degrade organic wastes, including rice straw, coconut endosperm, and palm waste, could be utilized by BSFL despite their high lignocellulose contents after pretreatment *via* biomass breakdown and fermentation (Caruso et al., 2014; Dickinson et al., 2019; Manurung et al., 2016; Mohd-Noor et al., 2017; Pamintuan, Agustin & Deocareza, 2020). Likewise, the degradation of high fiber substrates, such as palm kernel meal is recommended prior to use as a BSFL feed (Caruso et al., 2014).

A high waste reduction efficiency has been clearly observed after BSFL consumption. For example, BSFL could reduce 96.05% of 1.5 kg of fruit waste in 15 d, converting it into larval biomass at an 8.35% bioconversion rate (BCR) (Dzepe et al., 2021). Accordingly, BSFL could be a significant component of a “circular economy” to minimize both the consumption of agricultural resources and waste production in agri-food industries; whereby low value by-products that would normally be discarded are converted into insect protein-based foods (Leni, Caligiani & Sforza, 2021). In addition, the by-products of BSFL rearing (*e.g*., frass, chitin, and lipids) can be utilized with the goal of a “zero waste” industry (Chavez, 2021). Frass, which is composed of spent substrate, insect faeces, and exoskeleton, has the potential to be used as a biofertilizer to promote crop growth and induce disease resistance in plants (Leni, Caligiani & Sforza, 2021; Quilliam et al., 2020). Other insect-derived products also have the potential for research and industrial purposes, such as the production of fatty acids for biodiesel and cosmetic production, oleic acids for medicine production, and chitin for bioplastic production (Chavez, 2021; Leni, Caligiani & Sforza, 2021).

The use of SCG as an animal feedstock has rarely been studied (Givens & Barber, 1986), although there are some records of SCG utilization in dairy cattle, pig, sheep, earthworms, and BSF (Choi et al., 2018; Permana & Putra, 2018; San Martin et al., 2021; Sanchez-Hernandez & Domínguez, 2017; Sideris et al., 2021; Sikka & Chawla, 1986). Previous uses of SCG for BSF rearing have included as a larval diet (Fischer, Romano & Sinha, 2021; Permana & Putra, 2018; Sideris et al., 2021) and pupal substrate (Nakamura et al., 2016). As mentioned above, SCG-only substrates are not likely to be optimal for BSF growth, but rather they likely need a suitable pretreatment process to detoxify them and increase their digestibility. Previously, SCG has been fermented prior to use as a feed in order to degrade its toxic phenolic compounds and increase the quality of available nutrients (Ramírez, Pineda-Hidalgo & Rochín-Medina, 2021; Sanchez-Hernandez & Domínguez, 2017; Vítězová et al., 2019), but it has never been evaluated as a substrate for rearing BSFL. Lactic acid bacteria (LAB) fermentation by autochthonous *Lactobacillus plantarum* was found to be a potentially suitable process for improving the SCG availability in sheep feedstock preparation (Choi et al., 2018). In addition, the bacterial fermentation with *Bacillus clausii* released bioactive peptides from SCG *via* protein hydrolysates (Ramírez, Pineda-Hidalgo & Rochín-Medina, 2021). Thus, fermented (f) SCG produced by *Lactobacillus* spp. is a potential feed supplement for BSF.

Although BSFL can grow on various kinds of substrates, not all substrates can support a high larval growth performance. The protein: carbohydrate ratio in BSFL substrates is likely to be an important factor that influences both the larval growth and causes variation in the nutritional composition of the larval body (Barragán-Fonseca et al., 2018b). Based on the nutritional requirements of BSFL, high-quality substrates with 21% (w/w) protein and 21% (w/w) carbohydrate often result in larvae that develop faster, to a larger size, and with a higher survival rate (Laganaro, Bahrndorff & Eriksen, 2021; Leni, Caligiani & Sforza, 2021). It is difficult to find the perfect pure food substrate for BSF rearing. Instead, co-digest wastes have given a better growth of BSFL, especially for difficult-to-degrade organic wastes (Pamintuan, Agustin & Deocareza, 2020; Sideris et al., 2021). As a consequence, the combination of various organic wastes has been evaluated as potential high-quality and low-cost BSFL diets (Gold et al., 2020; Jucker et al., 2017). Thus, SCG has recently received interest as a low-cost dietary supplement for BSFL rearing, as mentioned above.

It is already known that the non-fermented SCG is not a suitable diet for BSFL (Fischer, Romano & Sinha, 2021; Permana & Putra, 2018; Sideris et al., 2021), but fSCG has never been evaluated as a feed or feed supplement for BSFL. According to Choi et al. (2018), *Lactobacillus* spp. can improve the protein digestibility of SCG when used in sheep feedstock. Thus, this study aimed to evaluate use of fSCG at different proportions as a supplement in a fruit-vegetable pulp feed for BSFL rearing in terms of the growth, development, mortality, and conversion efficiency.

## Materials and Methods

### Laboratory rearing of BSF

The BSF were cultured from a stock colony at the Department of Entomology, Kasetsart University, Bangkok, Thailand. The colony was established from eggs purchased from a farmer in the Khon Kaen province. The BSFL and adults were fed on a by-product of Thai rice noodles and brown sugar solution, respectively. The larvae were provided mixed fruits and vegetables from the Royal Project Foundation, whose products are certified organic by domestic and international food safety standards. The insects were maintained for 1 year (since 2000) under natural ambient conditions (approximately 28–34 °C, 70–90% RH, and 13:11 L:D cycle). The adults were maintained in a mesh room (4 m (L) × 3 m (W) × 3 m (H)), with a partially transparent roof and an automatic mist spraying system (1-min sprays, six times daily) to reduce the ambient temperature. The adults were fed a 30% (w/v) brown sugar solution. Egg clutches were laid in small gaps between wooden sheets above a container of mixed wheat bran, fruits, and vegetables, which acted as the oviposition substrate and attractant. One day before egg collection for the experiment, the eggs in the stock culture were removed to ensure synchrony in larval age (all 12-d-old at the start) in the experiment. Briefly, the egg clutches were gently removed from the wood surface using a cutter, and then 0.5 g of eggs were transferred to a hatching container containing baby food comprised of 1 kg corn meal and 4 kg pulp residue from a juice extractor. A total of 7,200 12-d-old larvae were prepared.

### Experimental design

Two organic wastes were used. The first was a pulp residue composed of (all (w/w)) apple (12%), pineapple (12%), carrot (25%), tomato (44%), guava (5%), beetroot (1.5%), and celery (0.5%) pulp, collected from a juice extractor at the Kasetsart University shop. All vegetables and fruits were washed twice under running tap water. The second was the SCG obtained from Jankahom’s café, a coffee shop in Bangkok. In our first trial, the preliminary results of feeding fresh SCG revealed very high larval mortality rates, which was probably due to the toxicity and low digestibility of SCG. As a consequence, the SCG was fermented prior to use as a feed in order to degrade its toxic phenolic compounds and increase the quality of available nutrients. Based on Choi et al. (2018), the SCG was probably digested during the fermentation by LAB, such as the autochthonous *L. plantarum*. Generally, *L. plantarum* and *L. rossiae* can be isolated from traditional fruit fermentations, particularly pineapple fermentation, without the need for additional nutrient supplementation or pH adjustment (Di Cagno et al., 2010; Garcia et al., 2020). Homemade, bio-fermented pineapples were then selected due to their ready availability and simple preparation, as this would be beneficial for BSF farmer extension programs. Prior to use, the SCG were mixed with pineapple-fermented water (PFW; 3:1 (w/w) pineapple peels: brown sugar with a more than 30-d incubation period (Tanganurat, 2012)) containing 3,300 cfu/mL *Lactobacillus* spp. to inoculate autochthonous LAB for the degradation of the SCG and the reduction of its phenolic compounds. The PFW was added to an anaerobic digestion of the substrate mixtures, a 2:¼:1:¼ (w/w) ratio of SCG: 5% PFW: brown sugar: water for 60 d in closed-lid bins at room temperature.

The treatments consisted of a mixture of the fruit residues and six different fSCG percentages by wet weight (w/w): 0% (control or only pulp residue), 20%, 40%, 60%, 80%, and 100% (w/w) of the larval diet. Six replicates were prepared to allow for the effect of handling disturbance. Prior to use, water was added to the 60–100% (w/w) fSCG treatments to adjust the substrate moisture to within the suitable range for BSF (>60%). The larvae were kept in plastic containers (Ø 24.5 × 12.5 cm), with ventilation lids covered with a mosquito net to prevent contamination by other insects. The diet amounts were calculated for 14 d at a feed rate of 200 mg/larva/d, following Permana & Putra (2018). In total, 560 g of diet was initially provided for each treatment. In the 40%, 60%, 80%, and 100% (w/w) fSCG diet groups, the larvae did not develop to prepupae within 14 d, so 120 g of additional diet was added on day 14 for the final 3 days of the experiment. Thirty larvae were randomly sampled from each group on day 1 and individually weighed on an analytical balance (OHAUS Pioneer PA214 Analytical Balance, USA) to get the initial weights of the tested larvae. The uniform size of the tested larvae was controlled in this process. Six replicates of 200 larvae (with a weight of 0.016 ± 0.003 g/larva) were hand counted and placed on the different treatment diets. However, the 100% (w/w) fSCG was examined using only one replicate.

### Growth performance of BSFL

All insect larvae were gently handled with a camel hair brush during counting and weighing to minimize disturbance to their development. Ten larvae from each treatment were randomly sampled and weighed every 3 days from replicate one to quantify larval growth until the pupal stage was reached. After weighing, the larvae were returned to their containers to continue development. To determine the effect of the different substrates on *H. illucens* development and weight, a sample of 30 mature larvae, prepupae, and pupae were randomly collected and weighed from replicates two–four to avoid any effects on larval development from handling, as this has frequently been observed in previous larval growth investigations. The mature larvae were sampled from the creamy white larvae at the time of 40% prepupal appearance. The prepupal weight was collected from larvae characterized by the presence of a black larval cuticle at 80% prepupal appearance. The occurrence of the first and last prepupae stages from each treatment was recorded to determine the larval duration. As the post-feeding stage was reached, the prepupae were transferred to a 0.65-L plastic container that contained a rice husk pupation substrate, and then allowed to develop into pupae on this dry material and weighed. Once emerged, adults were counted daily, their sex was identified following Julita et al. (2020), and they were weighed individually. All weights were measured using the balance described earlier. Developmental times from the first to the last prepupation were recorded. The number of live prepupae from three separate replicates (replicates 4–6) were counted to calculate survivorship.

### Calculations and statistical analysis

To determine the efficiency of BSFL in consuming each substrate, the total amount of substrate added, the residue at the end of the experiment, and the final biomass (from all six replicates) were obtained as dry mass, and the waste reduction index (WRI), reduction rate, feed conversion rate (FCR), and BCR were calculated using Eqs. (1)–(4), respectively, (ur Rehman et al., 2017).

(1) $$\rm{WRI = (D/t) \times 100; \;where\; D = (W-R)/W}$$

(2) $$\rm{Reduction\; rate = [(W-R)/W] \times 100}$$

(3) $$\rm{FCR = substrate\; consumed \;(g)/BSF\; biomass \;(g) }$$

(4) $$\rm{BCR\; (\%) = [BSF \;biomass \; (g)/substrate\; consumed \;(g)] \times 100}$$

where *W* is the total amount of substrate (g) added during time t (time from beginning the experiment to first prepupal appearance) and R is the residue weight after BSF digestion (g).

The mean BSF weight (larvae, prepupae, pupae, and adult), substrate reduction index, FCR, and BCR in the different substrate groups were analyzed *via* one-way analysis of variance (ANOVA), followed by Tukey’s HSD for multiple comparison test. Independent sample t-tests (*P* < 0.05) were used to determine the significance of differences between male and female weights. Non-parametric one-way ANOVA (Kruskal–Wallis test) was performed to test differences in the sex ratio among the different fSCG treatments. The single replicate of 100% fSCG was not used in the statistical analysis. All statistical analyses were performed *via* SPSS, version 14 (Copyright SPSS for Windows; SPSS Inc., Chicago, IL, USA).

### Biochemical analysis of substrates, biomass, and residue

The dry mass of BSF and residue, including the proximate analysis of all diet substrates, were analyzed at the Animal Nutrition Laboratory, Department of Animal Science, Kasetsart University following AOAC (Horwitz, 2010). The nutritional content of all the substrates were evaluated in terms of (analytical determinations) the water (moisture), crude protein, crude lipid, crude fiber, and ash contents, and were calculated on a dry mass (DM) basis.

The biochemical composition of the BSF biomass was, however, not analyzed, due to the low experimental yield. As mentioned above, only one replicate of the 100% fSCG was used to record the other parameters; thus, there was no DM data of its biomass.

## Results

### Biochemical composition

The nutritional content of all substrates was calculated on a DM basis and are shown in Table 1. The ash and moisture content were noted to decrease with an increasing fSCG content. Lipids, proteins, and carbohydrates were high in the fSCG compared to the pulp residue; therefore, the nutritional contents were likely to be related to the fSCG quantity. Fiber was found to be highest in the medium fSCG range (40–60%). Finally, the protein: carbohydrate proportion in all the substrates ranged between 0.11–0.16.

**Table 1:** Nutritional content of the six tested substrates.

Nutritional content	Diet substrates with various %fSCG						Non-fermented SCG (100%)
	0%	20%	40%	60%	80%	100%	
Dry matter (%)	12.61	24.76	40.12	51.71	57.26	66.25	41.12
Crude protein (%)	1.58	2.83	3.58	4.45	4.70	4.90	5.85
Carbohydrate (%)	10.12	19.22	29.68	37.11	38.49	42.83	28.21
Crude lipid (%)	0.49	1.94	3.24	3.80	4.37	5.42	6.34
Crude fiber (%)	2.87	3.28	4.05	3.69	2.97	2.34	4.53
Ash (%)	1.14	1.04	0.94	0.80	0.74	0.59	0.72
Protein to carbohydrate ratio	0.16 (1:6)	0.15 (1:8)	0.12 (1:8)	0.12 (1:8)	0.12 (1:8)	0.11 (1:9)	0.21 (1:5)

### Growth performances and survival

**Figure 1:**
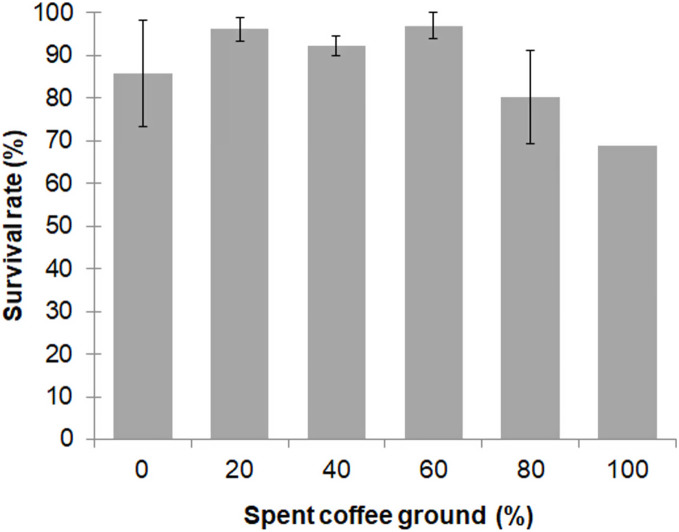
Survival rate of BSFL (until prepupae) reared a fruit-vegetable pulp diet supplemented with various proportions of fSCG. Data are shown as the mean ± 1SD, derived from three independent trials except for 100% fSCG, which is derived from a single trial. There were no significant differences between means (One-way ANOVA *F* = 2.557; *df* = 4, 10; *P* = 0.104).

**Figure 2:**
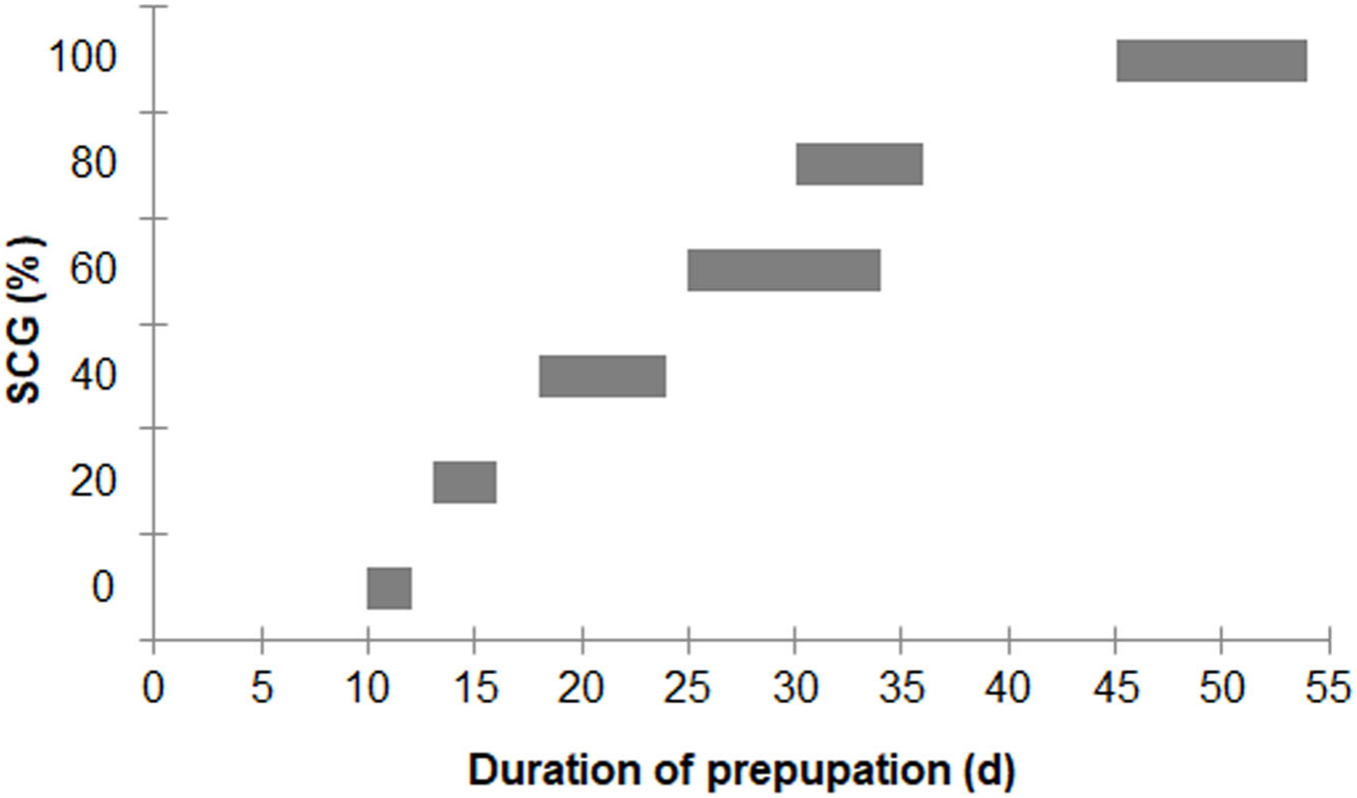
Ranges of prepupation duration of BSF reared on a fruit-vegetable pulp diet supplemented with various proportions of fSCG. Note, the duration (day) does not include the first 12 days from hatching (12-d-old BSFL were used at the start of the experiment).

Larval survival between the different diet groups was not significantly different (*F* = 2.557; *df* = 4, 10; *P* = 0.104), with an overall survival rate for the 0–80% fSCG diets of 90.3% ± 9.3%, although the 100% fSCG diet had the lowest survival rate (69.0%) (Fig. 1). Increasing the fSCG content negatively impacted on the developmental time, where higher fSCG content diets showed a more prolonged development time than those BSFL feeding on diets with a lower fSCG content (Fig. 2). With 100% fSCG, the larval duration was the longest (45–55 days).

**Figure 3:**
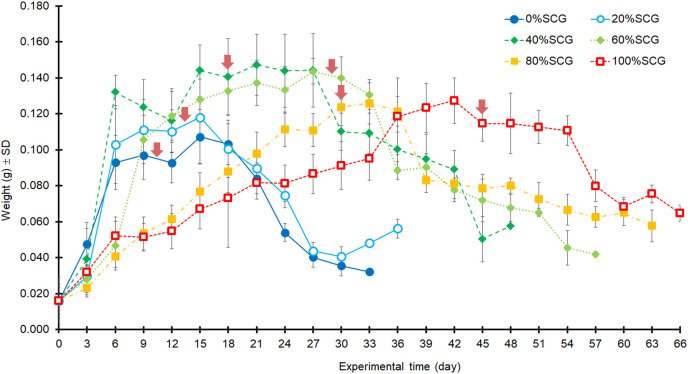
Growth curves of BSFL over time when reared on 200 mg/larva/d of fruit-vegetable pulp diet supplemented with various proportions of fSCG. Arrows indicate the first appearance of prepupa in each treatment. The duration (day) does not include the first 12 days of BSFL development (age) prior to starting the experiment. Data are shown as the mean ± 1SD, derived from 10 individuals per trial and three independent trials, except for 100% fSCG which is from one trial.

In addition, asynchronous development into prepupae was observed when using higher fSCG content feeds (40–100% fSCG), with the longest period of 9 d being observed with 100% fSCG. The fSCG contents affected the larval growth patterns (Fig. 3). During the first 6 days, differences in larval sizes appeared in the 0–40% fSCG (six- to eight-fold increase in weight), and the 60–100% fSCG (two- to three-fold increase in weight) groups. Subsequently, the 60% fSCG group doubled in weight by day 9. Meanwhile, the 80% fSCG and 100% fSCG diet groups required an extremely long period to develop into prepupae. In contrast, the larvae in the 0% fSCG (control) group developed rapidly with a similar growth rate as in the 20% fSCG group, followed by (in order) the 40–60% < 80% < 100% fSCG groups. The BSFL in the 40% and 60% fSCG groups had larger mean sizes (0.1472 and 0.1437 g, respectively) compared to the other treatments.

### Biomass of BSFL

The BSFL were supplied with 200 mg/larva/d of the different fSCG supplemented fruit-vegetable pulp diets and the BSF biomass was quantified from the wet weights of three different stages: larvae, prepupae, and pupae. Overall, decreasing sizes were noted from larvae into prepupae and then pupae (0.122 ± 0.017; 0.080 ± 0.013; and 0.068 ± 0.017 g, respectively). The use of fSCG in the diet increased the BSF wet weights in all three developmental stages (Table 2). The larvae and prepupae attained their highest wet weights in the 40–60% fSCG and 20–40% fSCG groups, respectively. All BSF stages displayed their highest wet weights in the 40% fSCG group. The weights of each stage were higher in the control group by 12.40%, 42.19%, and 18.46% for the larval, prepupal, and pupal stages, respectively.

**Table 2:** Weight of BSF larvae, prepupae, and pupae reared on a fruit-vegetable pulp diet supplemented with various proportions of fSCG. Data are shown as the mean ± 1SD. Means within a column with a different superscript letter are significantly different (*P* < 0.05).

%SCG	Larval weight (g)	Prepupal weight (g)	Pupal weight (g)
0%	0.121 ± 0.015^b^	0.064 ± 0.008^a^	0.065 ± 0.006^b^
20%	0.116 ± 0.013^b^	0.090 ± 0.008^c^	0.082 ± 0.008^c^
40%	0.136 ± 0.013^c^	0.091 ± 0.010^c^	0.077 ± 0.016^c^
60%	0.139 ± 0.010^c^	0.081 ± 0.007^b^	0.067 ± 0.010^ab^
80%	0.102 ± 0.068^a^	0.079 ± 0.009^b^	0.060 ± 0.006^a^
100%	0.117 ± 0.010^b^	0.074 ± 0.012^b^	0.060 ± 0.008^ab^
One-way ANOVA	*F* = 42.207; *df* = 5, 174; *P* = 0.000	*F* = 36.729; *df* = 5, 174; *P* = 0.000	*F* = 26.735; *df* = 5, 174 *P* = 0.000

### Adult weight and sex ratio

**Figure 4:**
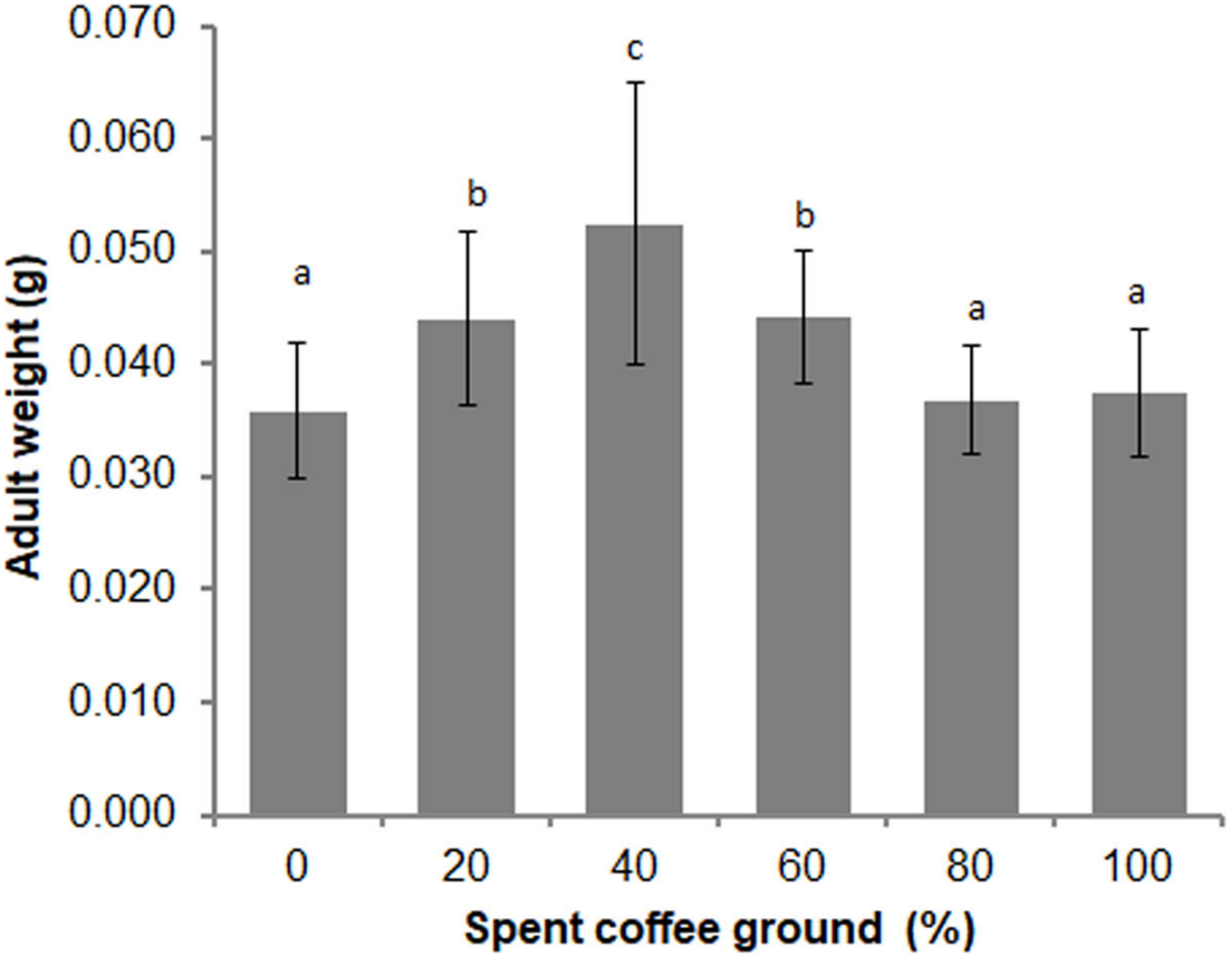
Comparison of the adult weight of BSF when reared on a fruit-vegetable pulp diet supplemented with various proportions of fSCG. Data are shown as the mean ± 1SD, derived from three independent trials *(*except for 100% fSCG, which is derived from a single trial). Means with a different letter are significantly different (*P* < 0.05).

Differences in the overall adult weight were observed between sexes (*t* = −2.346, *df* = 148, *P* = 0.020), with females weighing more than males (0.039 ± 0.007 g *vs* 0.043 ± 0.011 g, respectively). A significant difference in the adult weights among the various diets was also evident (*F* = 15.820; *df* = 5, 135; *P* = 0.000) (Fig. 4). The average adult weights in the 40% fSCG group was the highest (0.052 ± 0.013 g), followed by those in the 20% fSCG (0.044 ± 0.008 g), and 60% fSCG (0.044 ± 0.006 g) groups, while the 0% fSCG control (0.036 ± 0.006 g) and higher fSCG content groups (80–100%) were the smallest in size. The overall sex ratio (female: male) ranged from 0.60–1.23 and was not significantly different between treatments (Kruskal–Wallis test = 2.484, *df* = 4, *P* = 0.648) (Table 3). However, the higher fSCG content groups (80–100%) showed numerically male-biased sex ratios.

**Table 3:** Female-to-male (F: M) ratio of BSF adults reared on a fruit-vegetable pulp diet supplemented with various proportions of fSCG. Data are shown as the mean ± 1SD from three independent trials and the indicated number of adult flies in total.

fSCG	0%	20%	40%	60%	80%	100%
Sex ratio	1.1 ± 0.1	1.0 ± 0.1	1.0 ± 0.2	1.1 ± 0.2	0.9 ± 0.2	0.8
(F:M)						
Adult no.	515	577	554	578	418	130

### Waste reduction efficiency, BCR, and FCR

**Figure 5:**
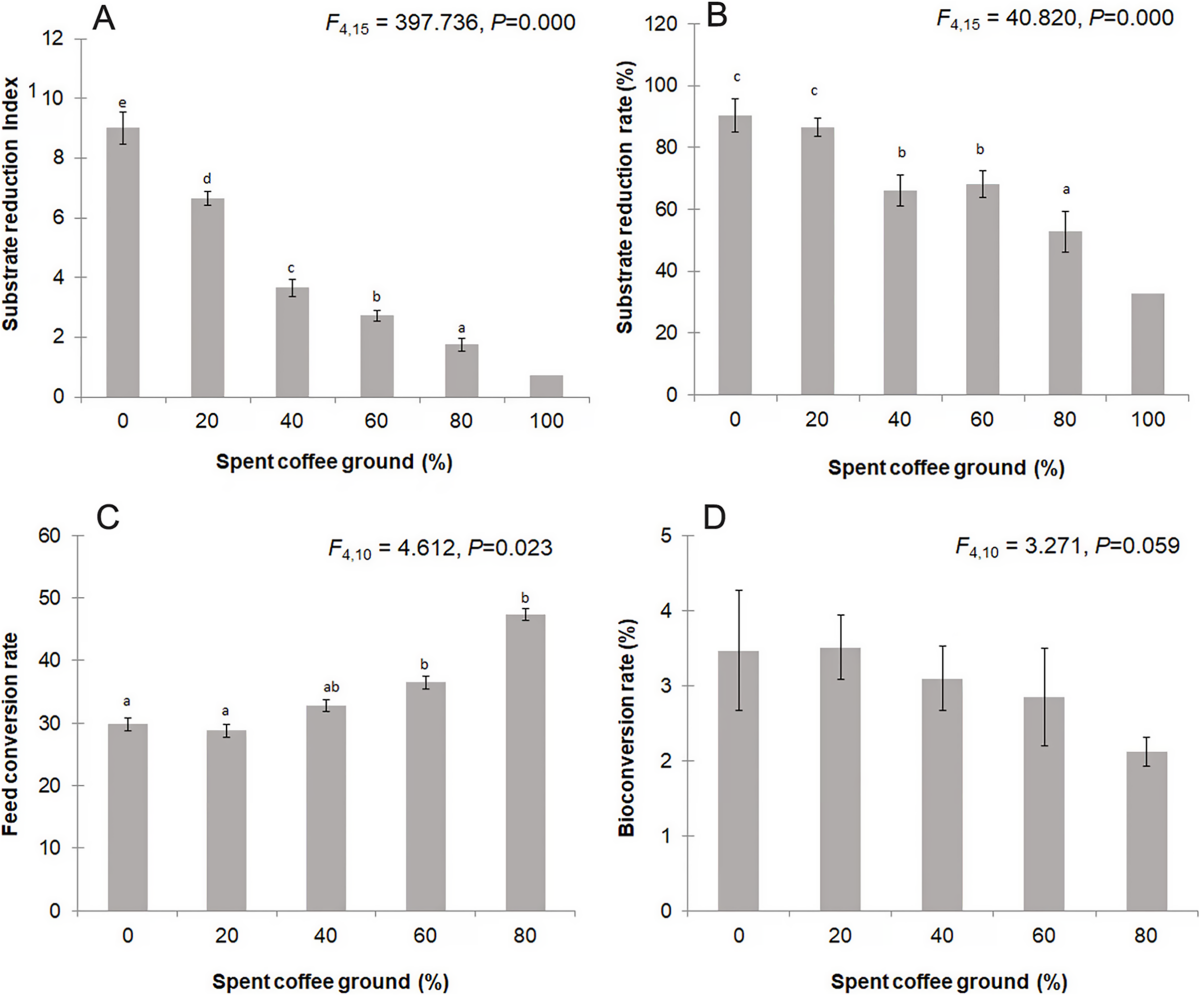
Comparison of the (A) substrate reduction index, (B) substrate reduction rate, (C) feed conversion rate, and (D) bioconversion rate by BSFL. Data are shown as the mean ± 1SD, derived from four independent trials, and means within a different superscript letter are significantly different (*P* < 0.05) except for 100% fSCG, which is derived from a single trial.

Figure 5 displays the waste reduction efficiency, FCR, and BCR of BSFL reared on the mixed fSCG substrates. The substrate reduction index (Fig. 5A) showed significant variation with different fSCG quantities (*F* = 397.736; *df* = 4, 15; *P* = 0.000), ranging from 1.55 to 9.68 (average ± SD, 4.67 ± 2.84). The reduction indices were highest in the substrate without fSCG (average ± SD, 9.02 ± 0.53), followed by that with 20% fSCG (average ± SD, 6.65 ± 0.22). These results were similar to those for substrate reduction rate (90.22% ± 5.33% and 86.47% ± 2.89%, respectively) (Fig. 5B). The FCR (Fig. 5C) of the 0–20% fSCG treatments averaged 29.31% ± 4.62%, which was significantly lower than in the higher fSCG treatments (*F* = 4.612; *df* = 4, 10; *P* = 0.023). No significant differences among the BCRs were observed, but it seemed to numerically decrease with increasing fSCG contents (Fig. 5D).

## Discussion

As difficult-to-degrade products and mildly toxic wastes products, SCGs have been processed to reduce their toxicity *via* several methods, *e.g*., warm treatment, microbial biodegradation, and aerobic fermentation, and then evaluated for many alternative usages. The combination with other organic substrates through anaerobic digestion seems to properly digest the SCG composition resulting in an enhanced methane production (Kim et al., 2017; Orfanoudaki et al., 2020). Microbial metabolism of fSCG can also decrease the caffeine, total polyphenols, and chlorogenic acid contents in wastewater treatment. In addition, bacterial fermentation with *B. clausii* was found to release bioactive peptides from the SCG as protein hydrolysates (Ramírez, Pineda-Hidalgo & Rochín-Medina, 2021). In the case of feedstock use, fermentation of SCG using *L. plantrum* improved its protein digestibility (Choi et al., 2018). This study selected a mixture of *Lactobacillus* spp. in the fermentation of SCG.

It was evident that the pure non-fermented SCG was not an optimum substrate for BSF growth, as established in previous research. However, whilst BSFL fed on pure SCG were previously reported to be unable to completely develop into pupae (Sideris et al., 2021), in this study they could develop to adults successfully, but at a very slow growth rate and with a slightly male-biased sex ratio. In accord, Permana & Putra (2018) found that pure unfermented SCG could support BSFL, but with slow growth rates, and increasing feed rates enhanced the development time. Although the small size and low survival rate (45.13% ± 10.2%) on pure non-fermented SCG substrate was demonstrated, the mixing of SCG with other food waste (dough) displayed surprisingly higher larval sizes, fast development, and high survival rates (81.16% ± 12.5%) (Fischer, Romano & Sinha, 2021). Similarly, a 1:1 (w/w) mixture of SCG: brewer’s spent grains or/and brewer’s yeast increased the BSF weight (Sideris et al., 2021). Likewise, the mixture of fSCG and pulp residue in this study demonstrated a better BSF growth performance than with SCG alone. In addition, the protein: carbohydrate ratio in the BSF diet was shown to be an important factor that needs consideration, where a 1:1 and 1:3 (w/w) ratio of protein: carbohydrate supported a high BSF growth performance, and was better than the 1:5 or 5:1 ratio (Barragán-Fonseca et al., 2018b; Cammack & Tomberlin, 2017; Gold et al., 2020). Correspondingly, 0–20% fSCG in this study could support the larger sizes of BSFL.

The mixture of different substrate resources might help balance the nutritional content of the diet. As mentioned above, BSFL likely need a 25–50% (1:1 to 1:3) protein:carbohydrate ratio as a resource. Although adding fSCG in this study did not result in the perfect protein range, the fSCG substrate mixture increased the protein content two to three times, while the carbohydrate quantity was further increased two to eight times with the addition of the fruit residue. Thus, the BSFL weight when reared on the fSCG-supplemented substrates was larger than the control (no fSCG). These results indicate that the selection of other organic wastes with proper (suitable) nutritional contents (based on the C/N ratio) for fSCG co-substrates might be a good method to promote an improved performance of BSF rearing. In the case of fruit pulp residues, the juice was mostly separated, and so the pulp residue substrate had a low moisture content (60–70%), low nutritional contents, and smaller particle sizes. Its combination with fSCG is unlikely to be the ideal diet for BSFL, even though the larvae provided pure pulp residue (0% fSCG) and 20% fSCG showed very high substrate reduction rates (90.22% ± 5.33% and 86.47% ± 2.89%, respectively) and the highest BCR. As a consequence of the effect of the main substrate’s nutrition on the BSFL growth, the use of other food sources instead of pulp residue would likely yield different results. The mixting of fSCG with other higher nutritional substrates (at a crude protein and carbohydrate content of > 1.58% and > 10.12%, respectively) might support the growth of larger larvae in a shorter developmental time.

Generally, the adult body size was related to the sex, with larger females (Barragán-Fonseca, Dicke & van Loon, 2018a). The larval diet and density are important factors affecting adult sizes (Gobbi, Martínez-Sánchez & Rojo, 2013; Jones & Tomberlin, 2019; Miranda, Cammack & Tomberlin, 2019). At lower larval density and higher nutritional diet could produce heavier adults. The effect of the larval diet on the adult size was correlated to the wing, ovary, and basal oocyte sizes, the latter two of which might relate to the number of laid eggs (Gobbi, Martínez-Sánchez & Rojo, 2013). Thus, in the case of the production of adult flies, we do not recommend feeding BSFL with high concentrations of fSCG (>60%) in order to avoid obtaining small adults and male-biased sex ratios.

The high lignocellulose contents in the poorly or undegraded SCG could adversely influence the BSFL conversion rate, but the fermentation of SCG prior to use as a supplementary feed for rearing BSF has received considerably less attention. The process is not only necessary for caffeine and polyphenol degradation, but also for organic matter conversion. Recently, many studies have developed techniques to extract and isolate valuable compounds from SCG. Microbial fermentation has been found to improve the nutritional contents of SCG (Ramírez, Pineda-Hidalgo & Rochín-Medina, 2021; Sanchez-Hernandez & Domínguez, 2017; Vítězová et al., 2019). Additionally, SCG vermicomposting significantly increased the micro- and macro-nutrient levels and resulted in a neutral pH in the SCG, thus enhancing the earthworm density and biomass (Sanchez-Hernandez & Domínguez, 2017). The spontaneous fermentation of SCG by adding PFW was used in this study, as it is a convenient technique that can be followed by farmers, is safe for humans, and results in no biomagnification effects from chemicals. As a consequence of the larger-sized BSF obtained when using a fSCG feed supplement, it can be concluded that spontaneous fermentation is an effective pre-treatment of SCG. According to the substrate reduction of 32.88%, a 100% fSCG diet could be used for BSF ingestion; however, evaluation of other microbes or the use of integrated techniques will probably be more effective.

The results of this study indicated that fSCG has the potential as a feed supplement for rearing BSFL. The fermentation techniques still need additional study to further optimize (increase) the SCG digestibility to improve the nutrient availability, which should subsequently improve the development time and BSF yields. The conversion efficiency depends on the nutrition, moisture, and texture of the co-substrate organic wastes. The moisture content of the fSCG co-substrates needs to be considered due to the starving behavior and high mortality when not optimal, as discussed in Fischer, Romano & Sinha (2021). This may have been the cause of the low survival rate (45.13% ± 10.2%) in that study’s pure SCG treatment, due to the easy dehydration of the SCG structure. In this study, the substrate mixtures with high fSCG contents were observed to dry out easily, and needed to have water added to maintain the optimum 60–70% moisture range, but still gave a 69% survival rate in the 100% fSCG diet. The fine and dense texture might have caused the low survival rate in the high percentage fSCG mixtures, as these would result in a lack of aeration in the substrate. Thus, fibrous wastes were recommended as fSCG co-substrates.

Differences in the developmental growth and size were noted with each diet. The 20% fSCG-supplemented diet showed the best growth performance with short rearing times and large harvest sizes (prepupal stage), while the 40% fSCG diet supported the largest BSF size (larval and prepupal stages) but with longer rearing times. The cost of BSF production, a trade-off between the rearing time and BSF yield, is also important at a commercial scale.

This study evaluated the potential for fSCG to be used as a BSF feed supplement to utilize the abundant coffee waste found in urban areas. The BSF production is a potential urban waste management method that could change waste to insect mass. Further research should examine effective methods to increase the BSFL conversion efficiency on the fSCG substrate; for example, by using mechanical digestion methods or other kinds of microbial pre-treatments that result in an increased BSF biomass with a short rearing time. Interestingly, fSCG supplementation can help reduce the foul odor of substrate decomposition, which is an important problem in BSF farming in urban areas. The mechanisms of this should be examined in further studies.

## Conclusions

Due to its ability to consume various organic diets, BSFL can be effectively used to convert waste fSCG, which has a high nutritional content, as a dietary supplement to biomass. In this study, SCG was fermented to reduce its toxic contents and to increase its available nutrients prior to use as a BSFL feed supplement. We then examined the effect of different fSCG percentages in a fruit and vegetable pulp feed on the biological parameters and conversion efficiency of BSF. The results indicated that BSFL were able to survive on pure (100%) fSCG with high FCRs, but resulted in a male-biased adult sex ratio. A mixture of 20–60% fSCG in the fruit-vegetable pulp was found to give the highest yield of BSF, but higher fSCG percentages prolonged the development time. The larvae with the shortest development time, highest yield, highest survival rate, and highest conversion rate were found when reared on a 20% fSCG supplementation. The efficiency of fSCG conversion is probably related to the co-substrate; and the protein: carbohydrate ratio of the whole substrate mixture needs to be 1:1 to 1:3. Moreover, SCG fermentation requires further study to improve the SCG degradation prior to use as an insect feed to improve its nutrient availability. This information could then be applied to other hard-to-degrade wastes.

## Supplemental Information

10.7717/peerj.14340/supp-1 Supplemental Information 1 BSF growth performance data.

10.7717/peerj.14340/supp-2 Supplemental Information 2 BSF stock colony images.

## Additional Information and Declarations

### Data Availability

The following information was supplied regarding data availability: The raw measurements are available in the Supplemental File.
