# Supplementary material for: Use of fermented spent coffee grounds as a substrate supplement for rearing black soldier fly larvae, Hermetia illucens (L), (Diptera: Stratiomyidae)
Source: PeerJ. 2022 Oct 31;10:e14340. doi: 10.7717/peerj.14340 (PMC9632463; doi:10.7717/peerj.14340)
Supplement: Supplemental Information 2 [file peerj-10-14340-s002.pdf]

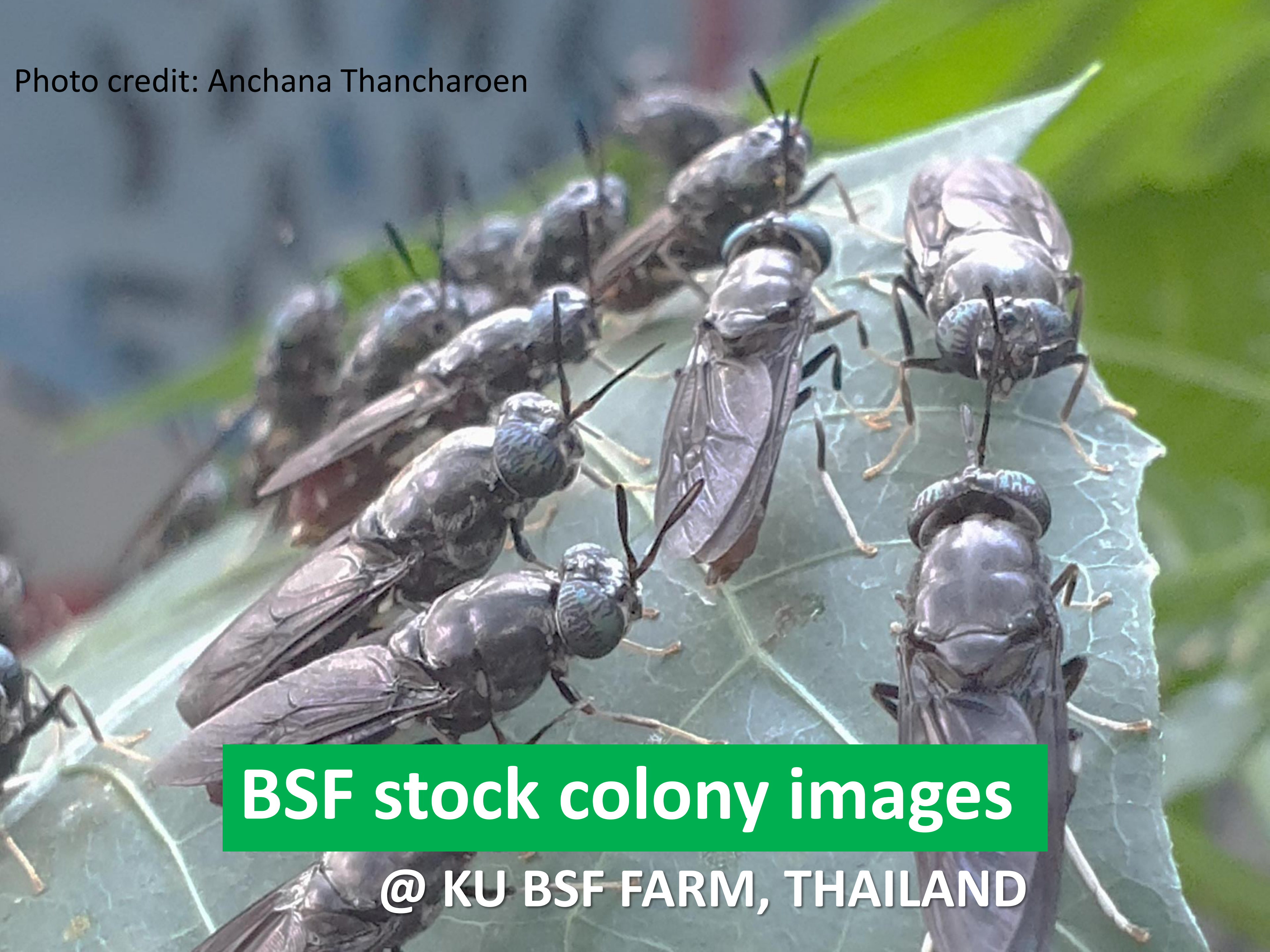
Photo credit: Anchana Thancharoen
BSF stock colony images
@ KU BSF FARM, THAILAND

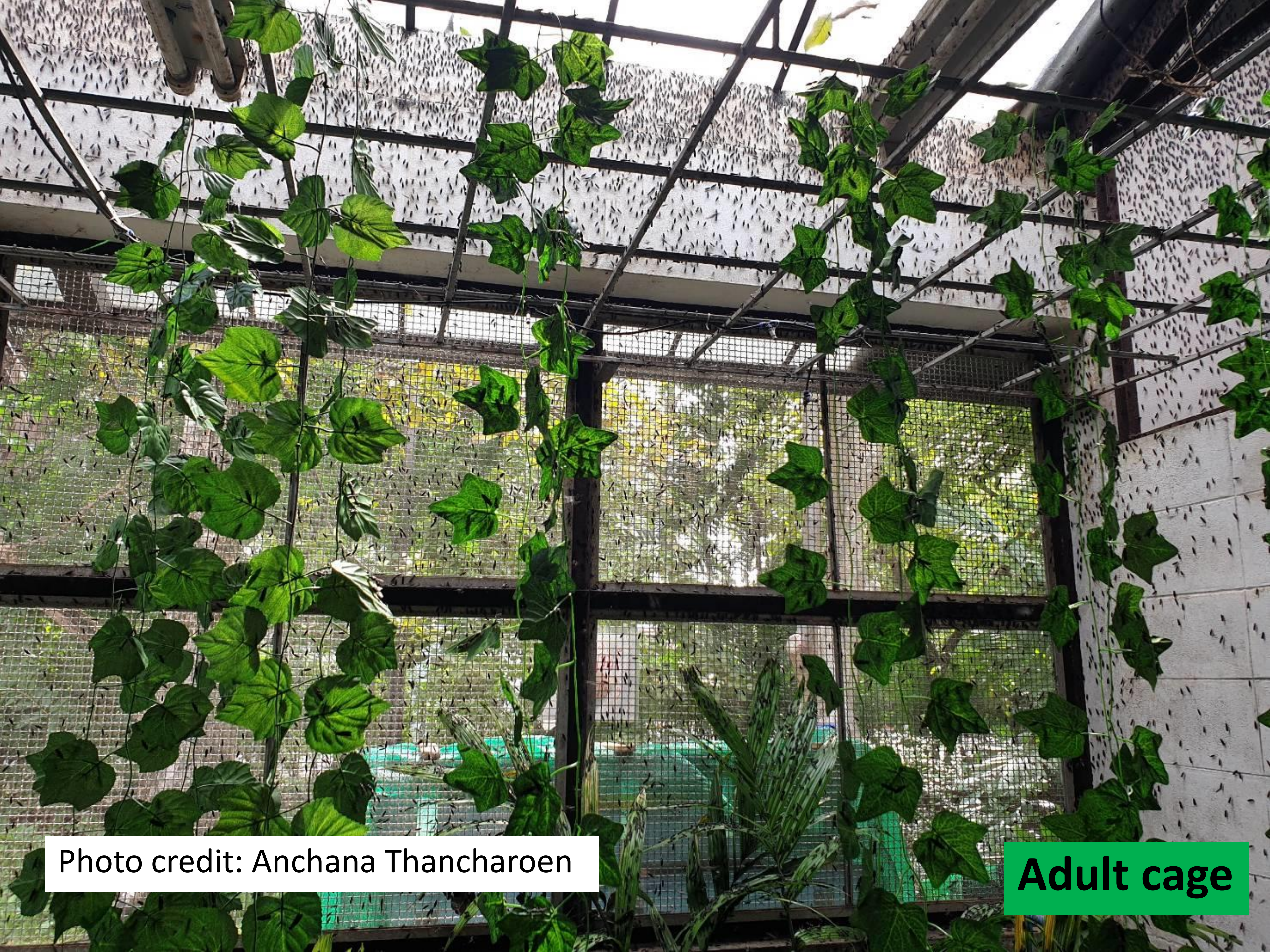
Photo credit: Anchana Thancharoen
Adult cage

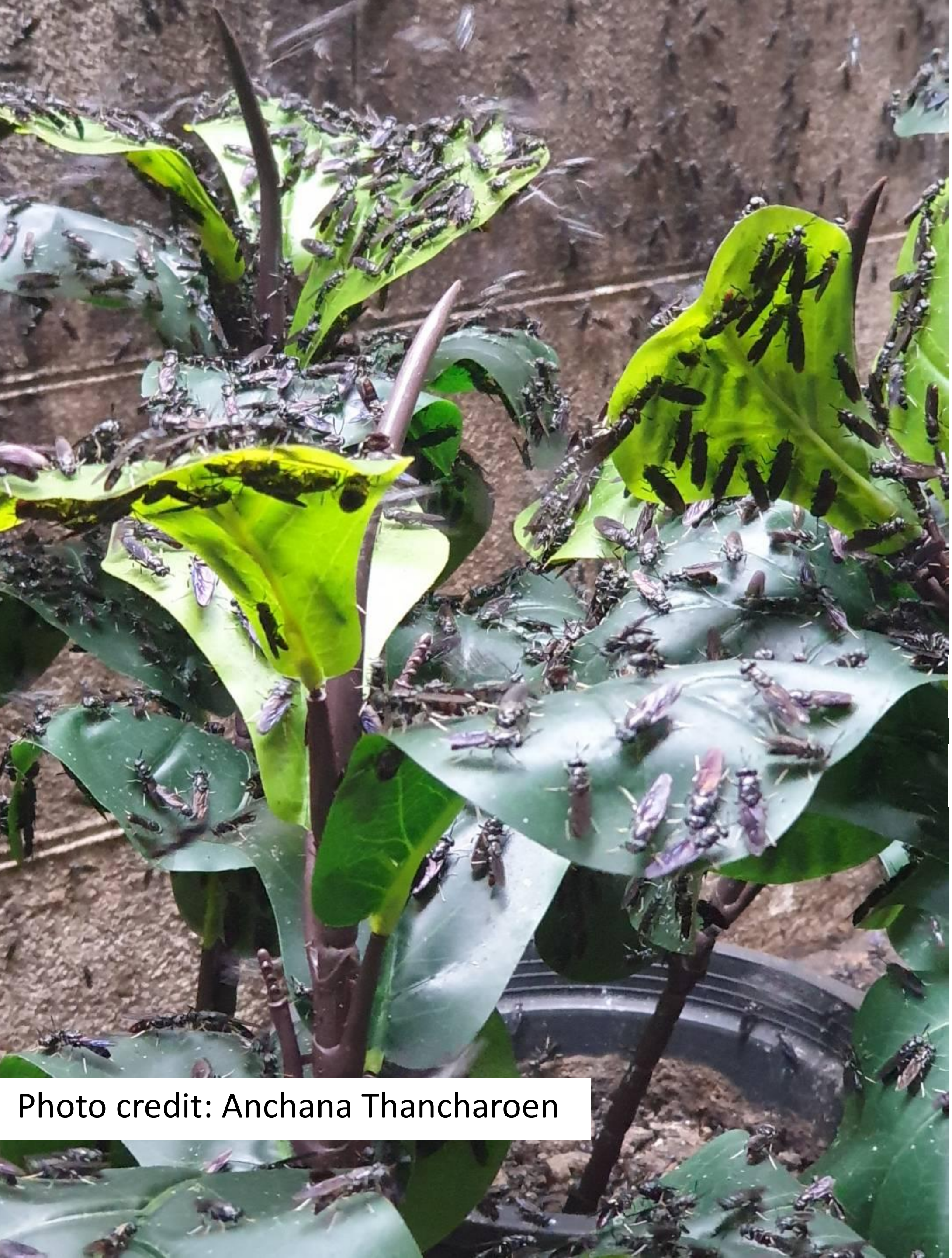

Photo credit: Anchana Thancharoen

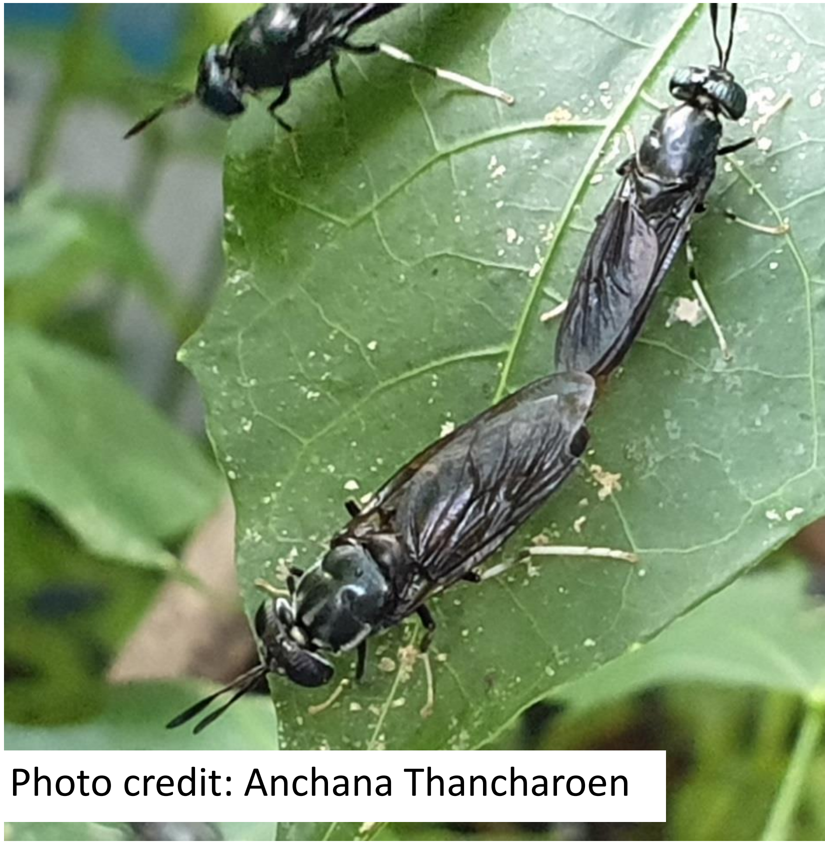

Photo credit: Anchana Thancharoen

Photo credit: Anchana Thancharoen

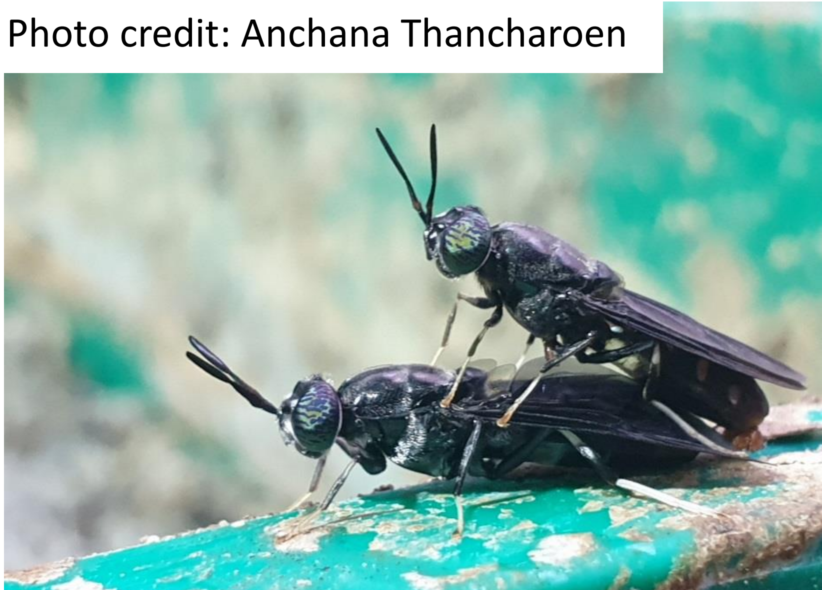

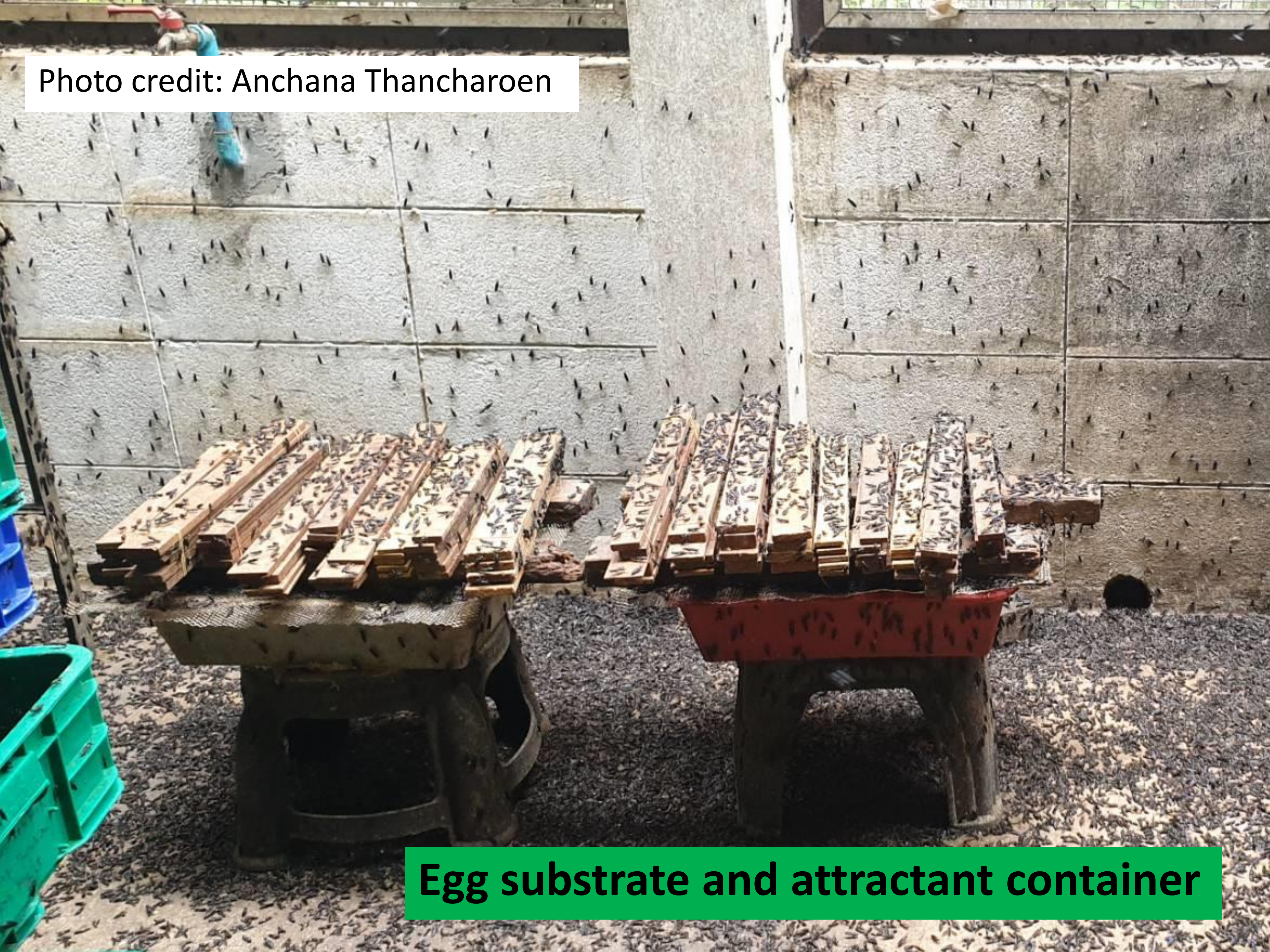
Photo credit: Anchana Thancharoen
Egg substrate and attractant container

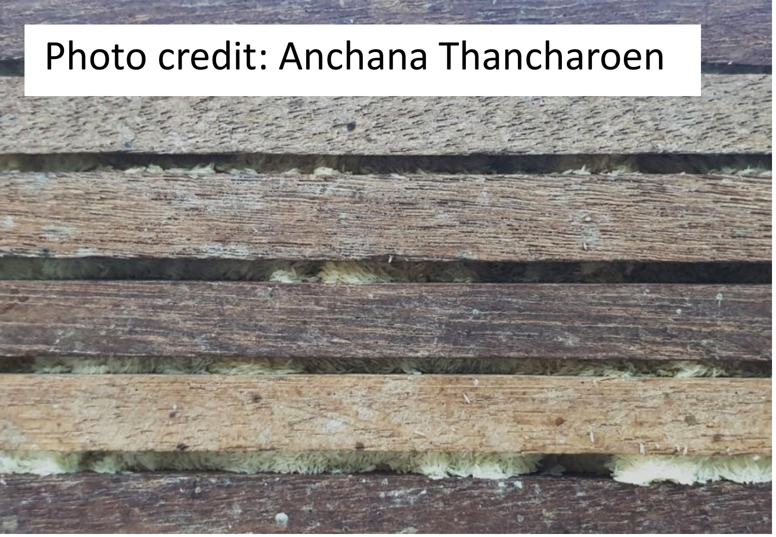
Photo credit: Anchana Thancharoen

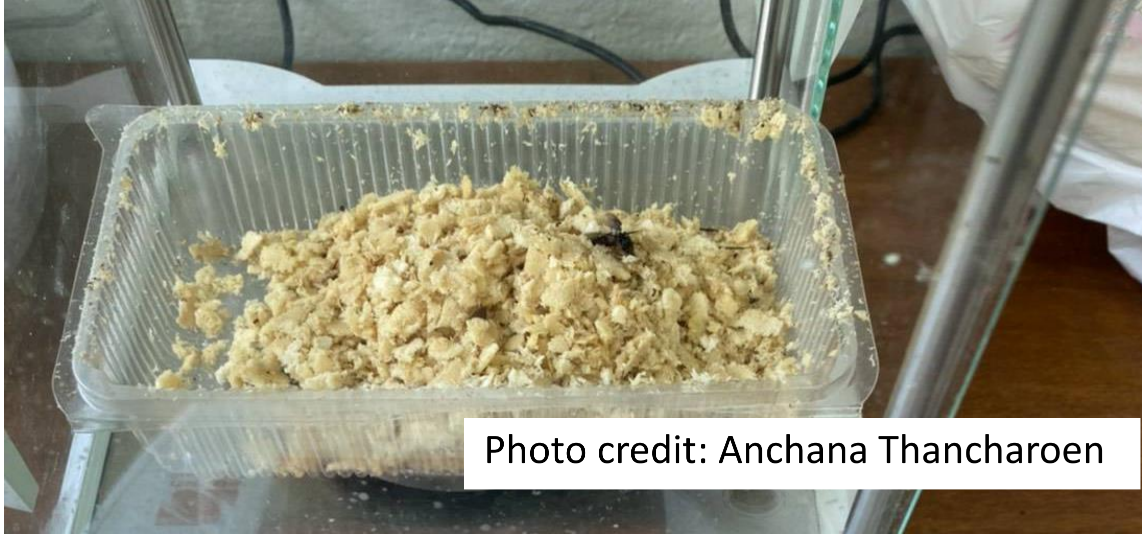
Photo credit: Anchana Thancharoen

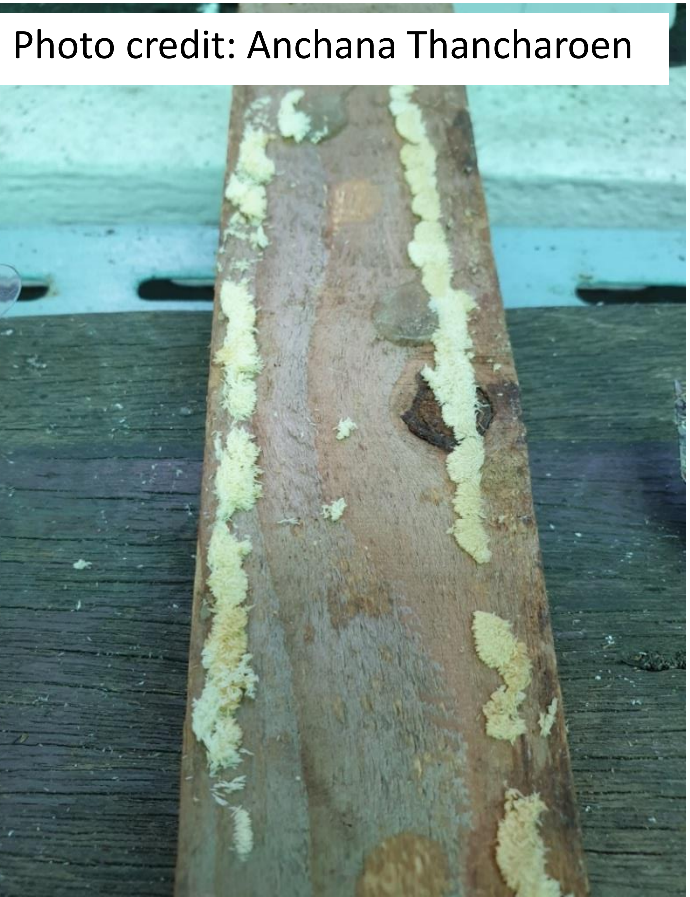
Photo credit: Anchana Thancharoen

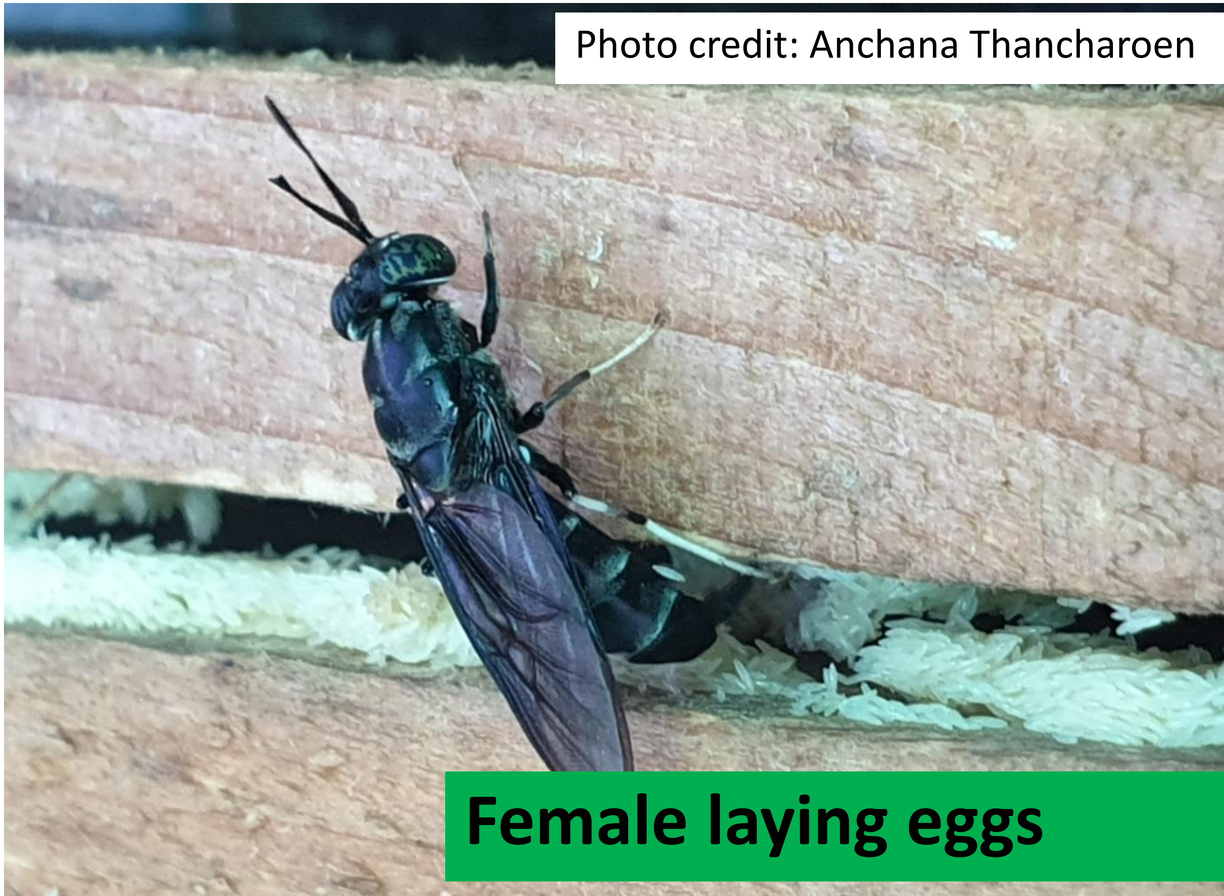
Photo credit: Anchana Thancharoen
Female laying eggs

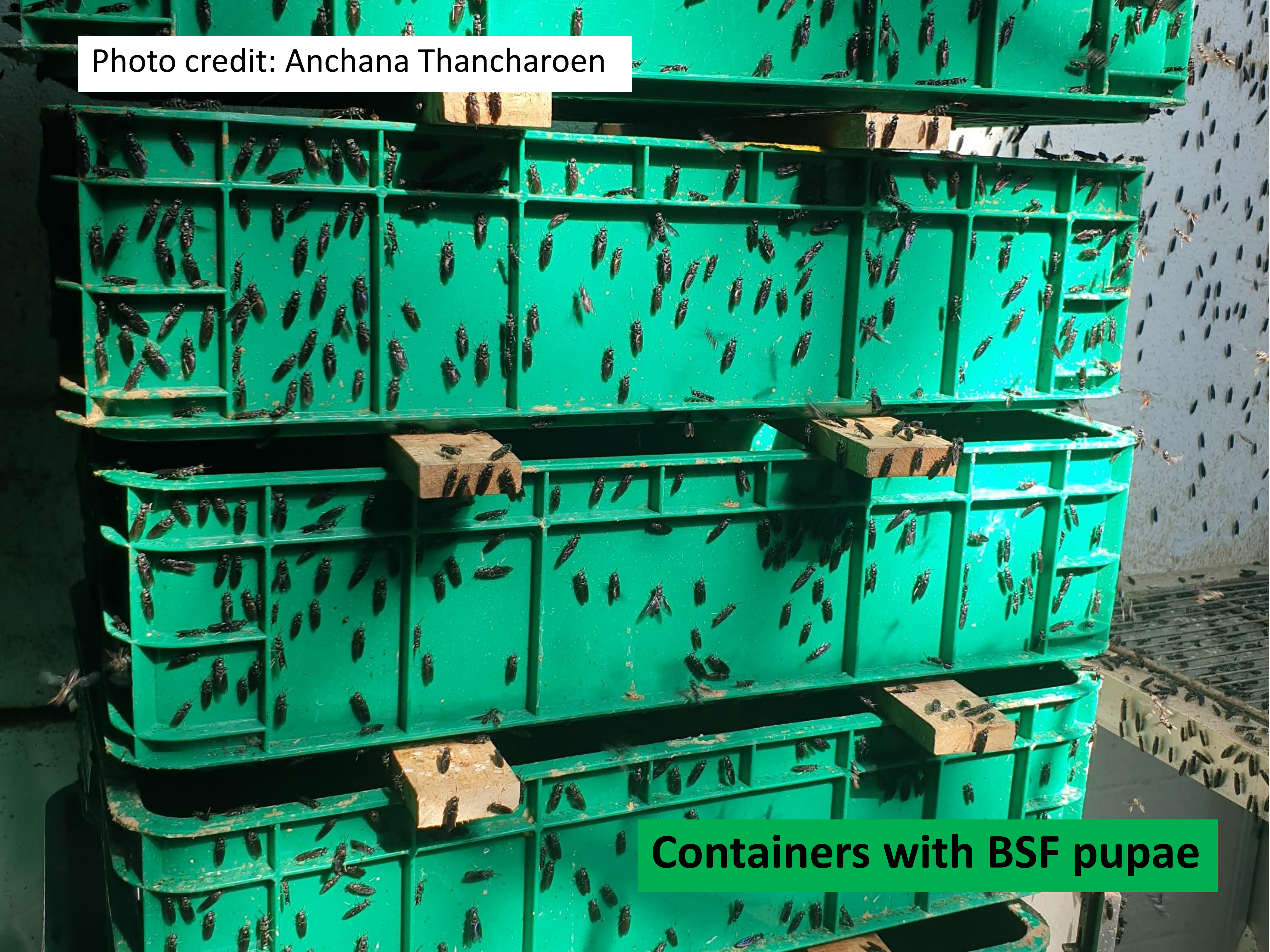
Photo credit: Anchana Thancharoen
Containers with BSF pupae

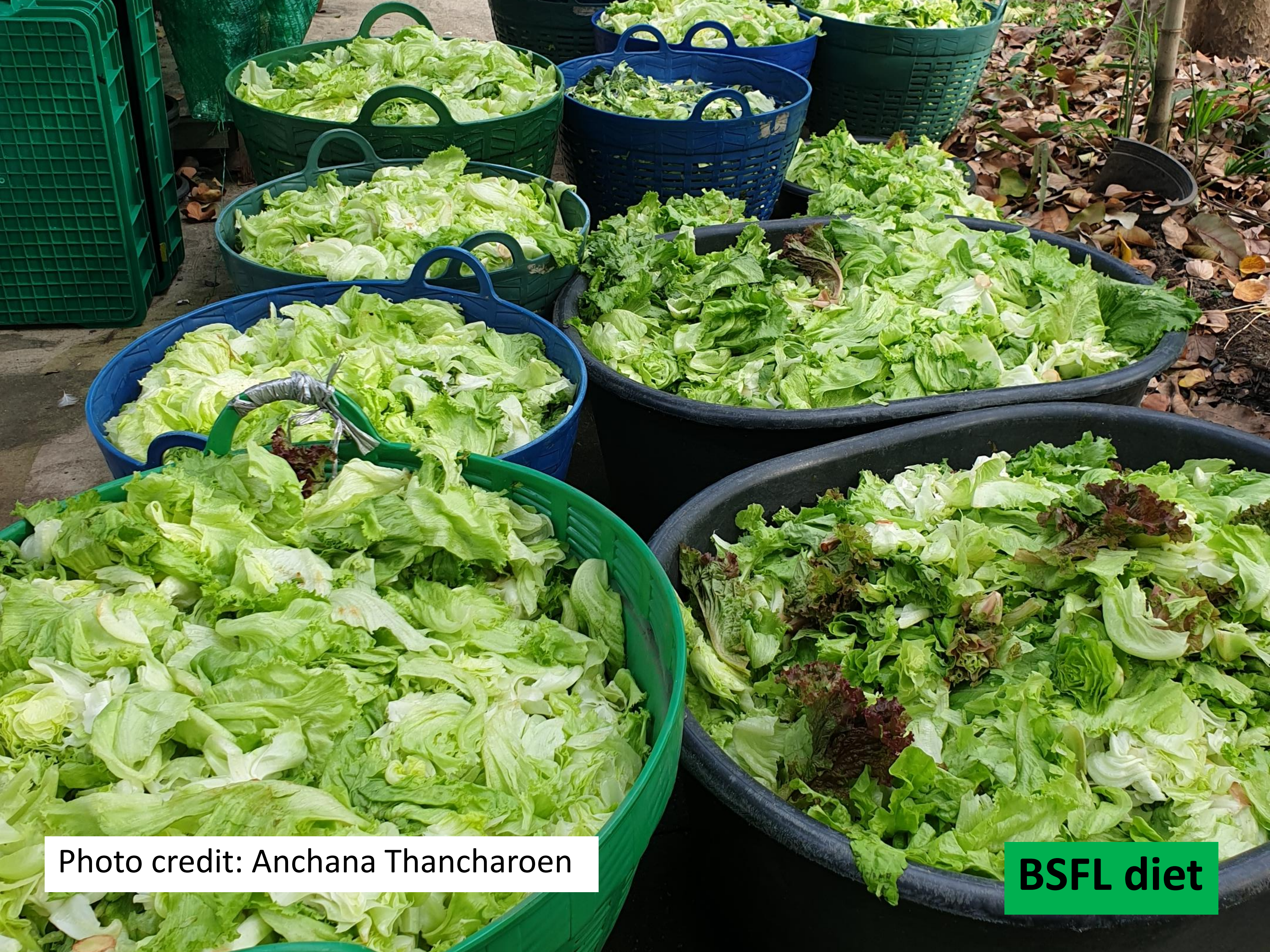
Photo credit: Anchana Thancharoen
BSFL diet

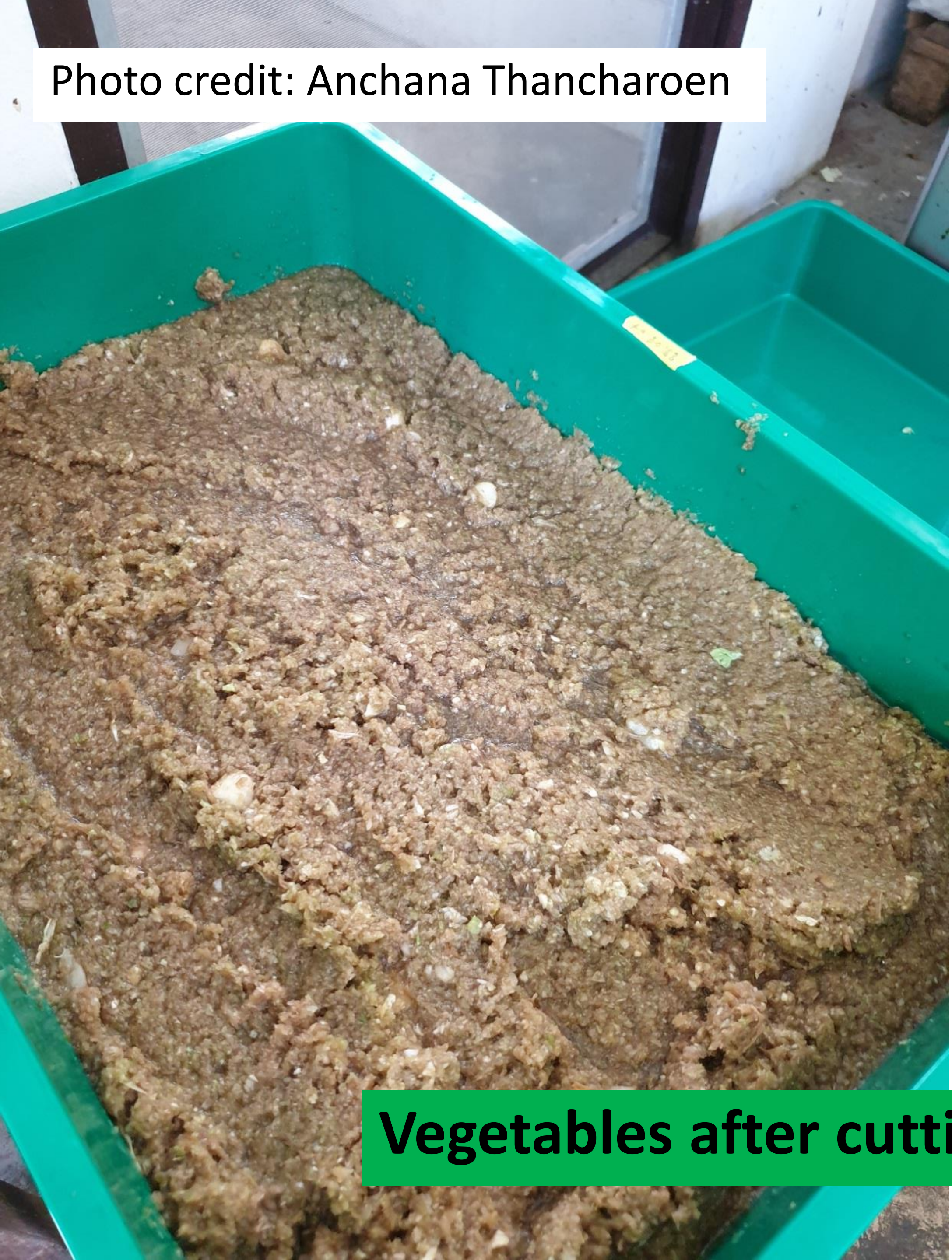

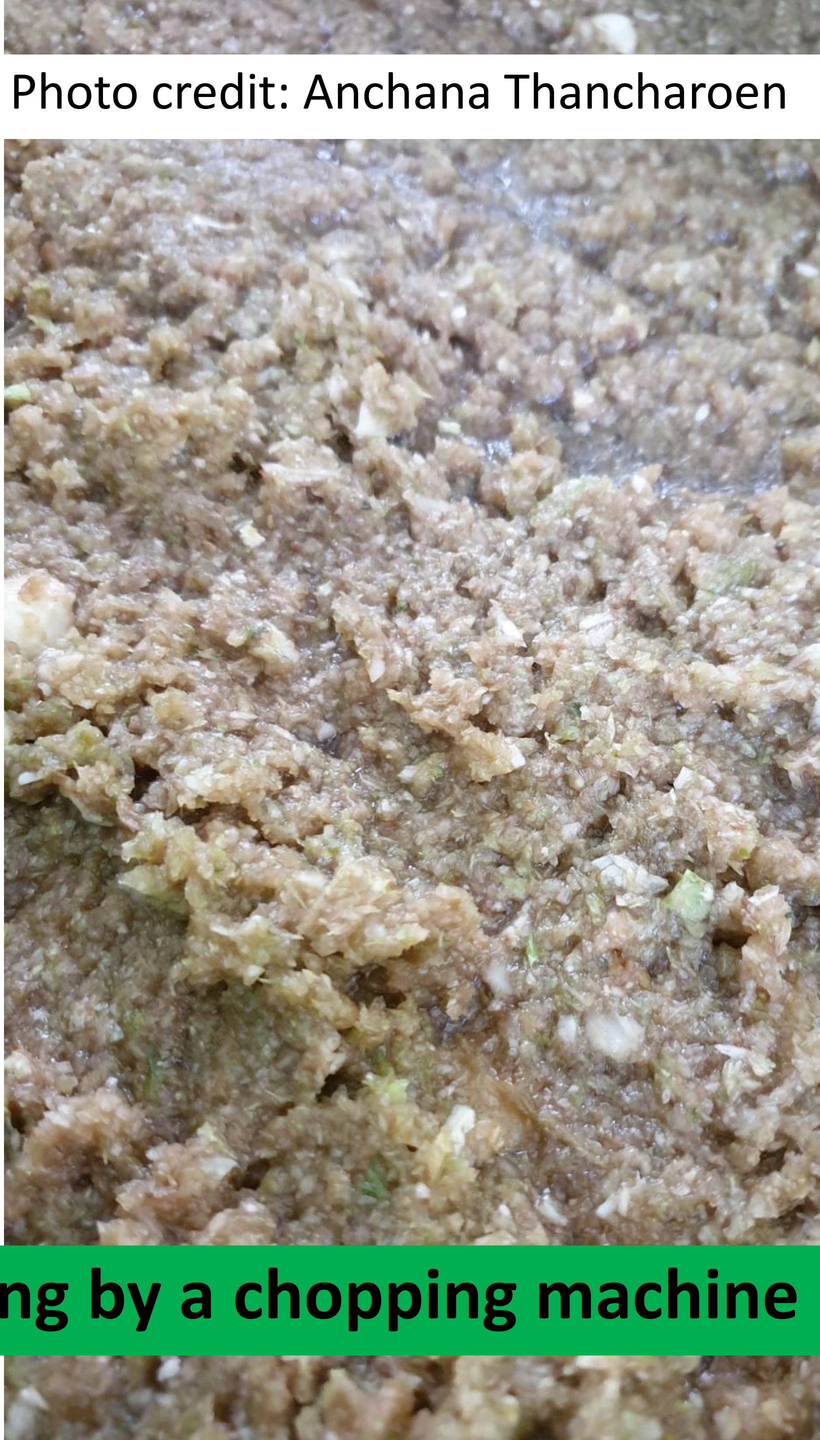

Vegetables after cutting by a chopping machine

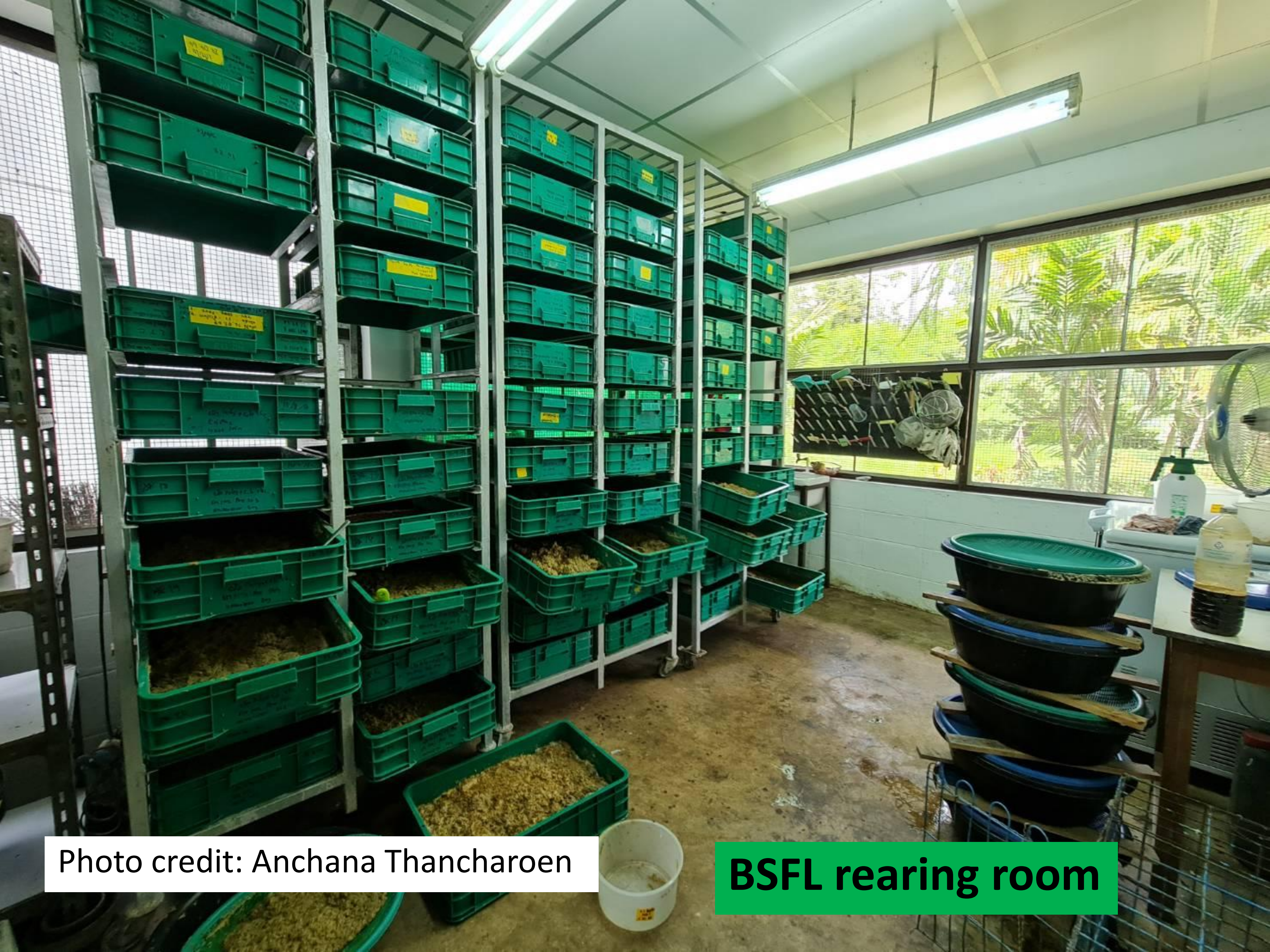

Photo credit: Anchana Thancharoen

BSFL rearing room

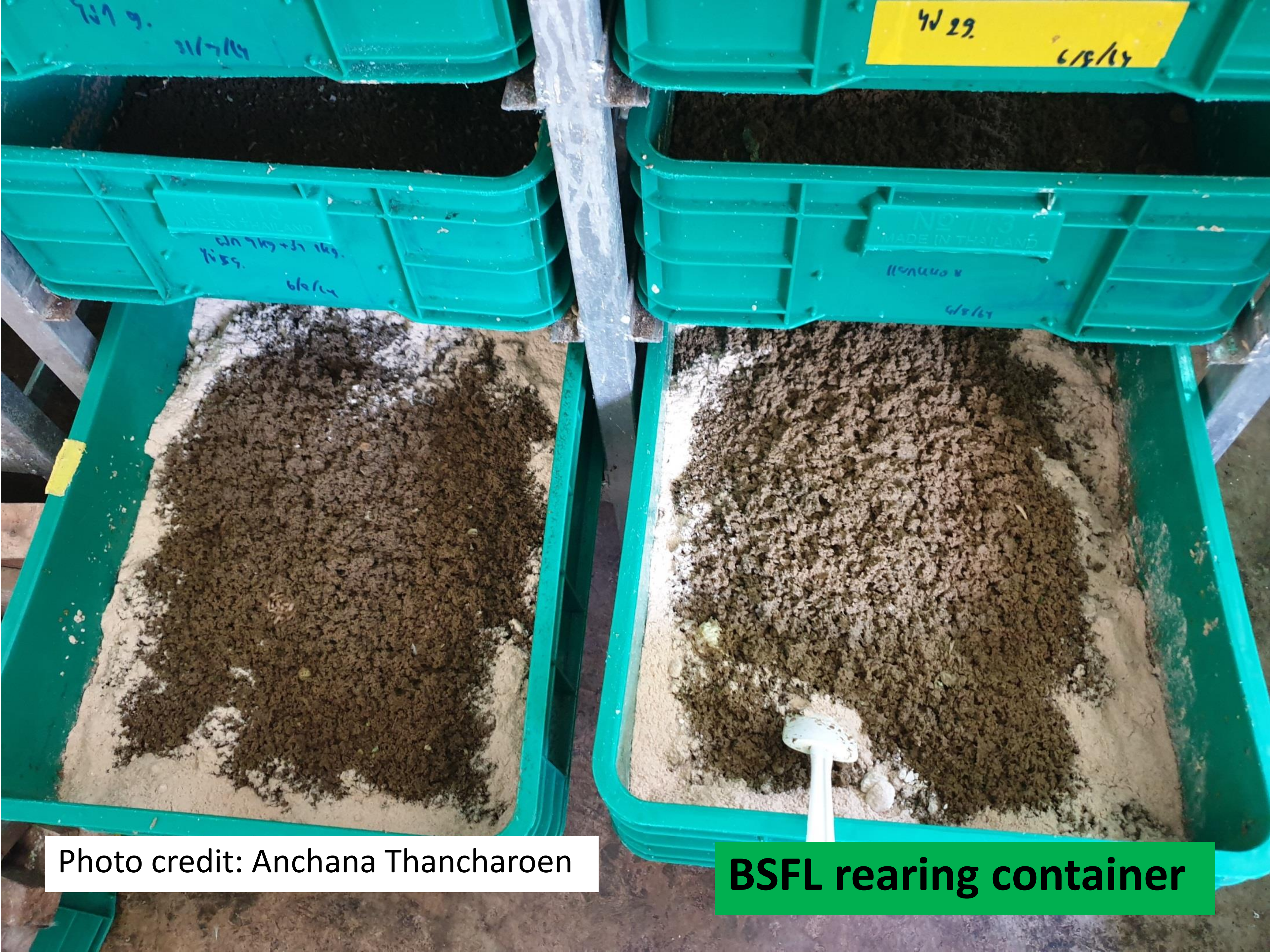
Photo credit: Anchana Thancharoen

BSFL rearing container

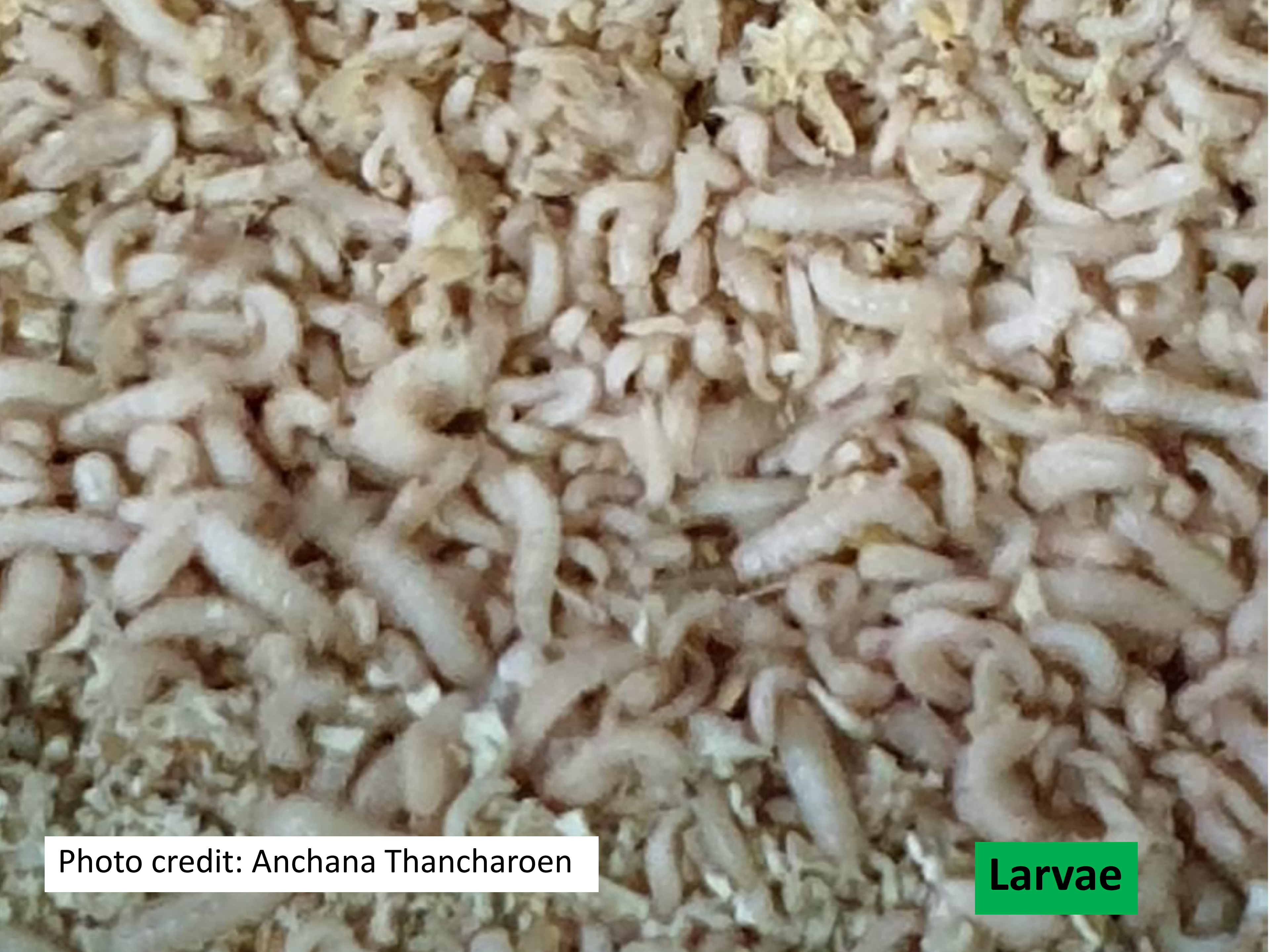
Photo credit: Anchana Thancharoen
Larvae

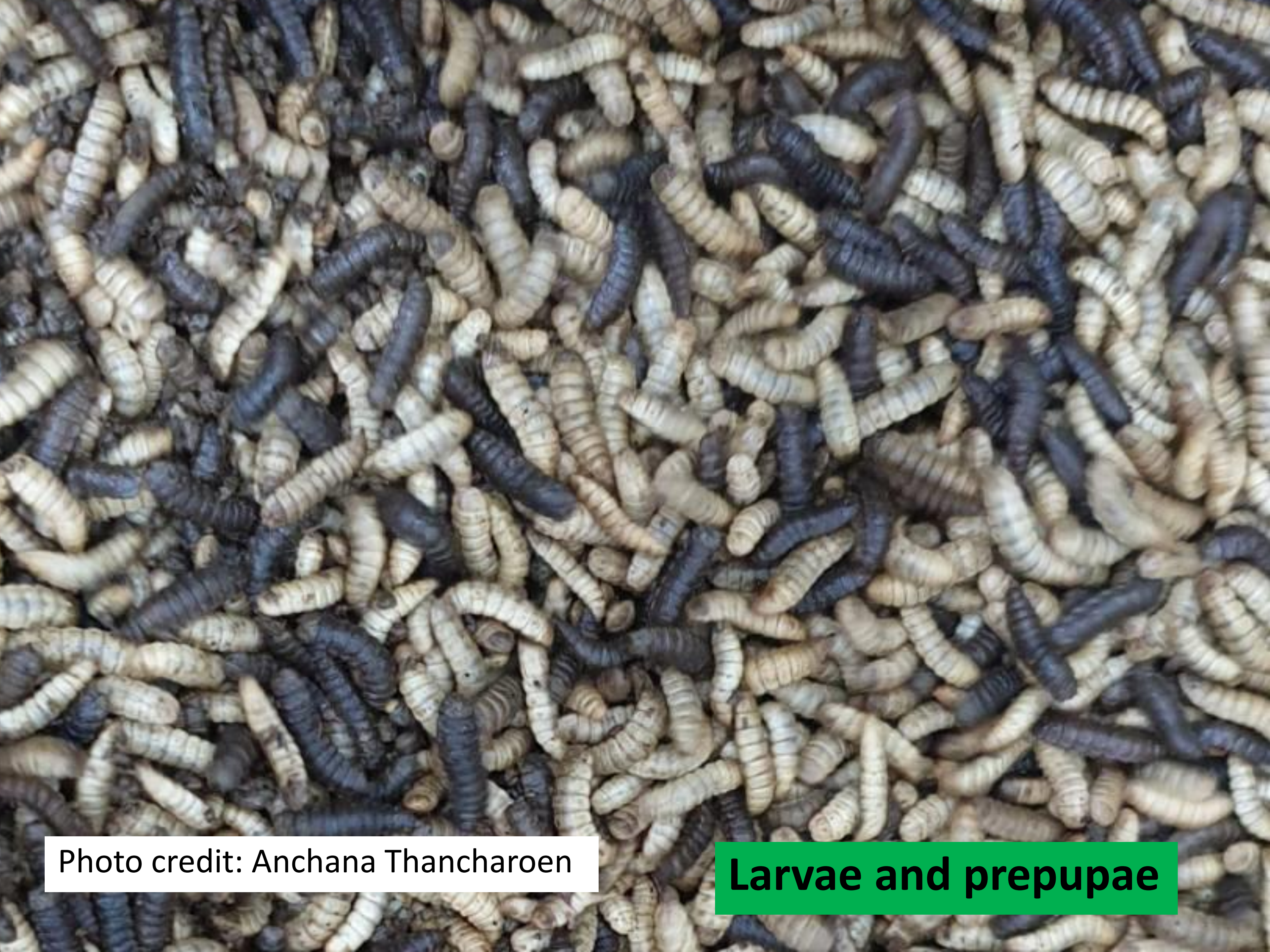

Photo credit: Anchana Thancharoen
Larvae and prepupae

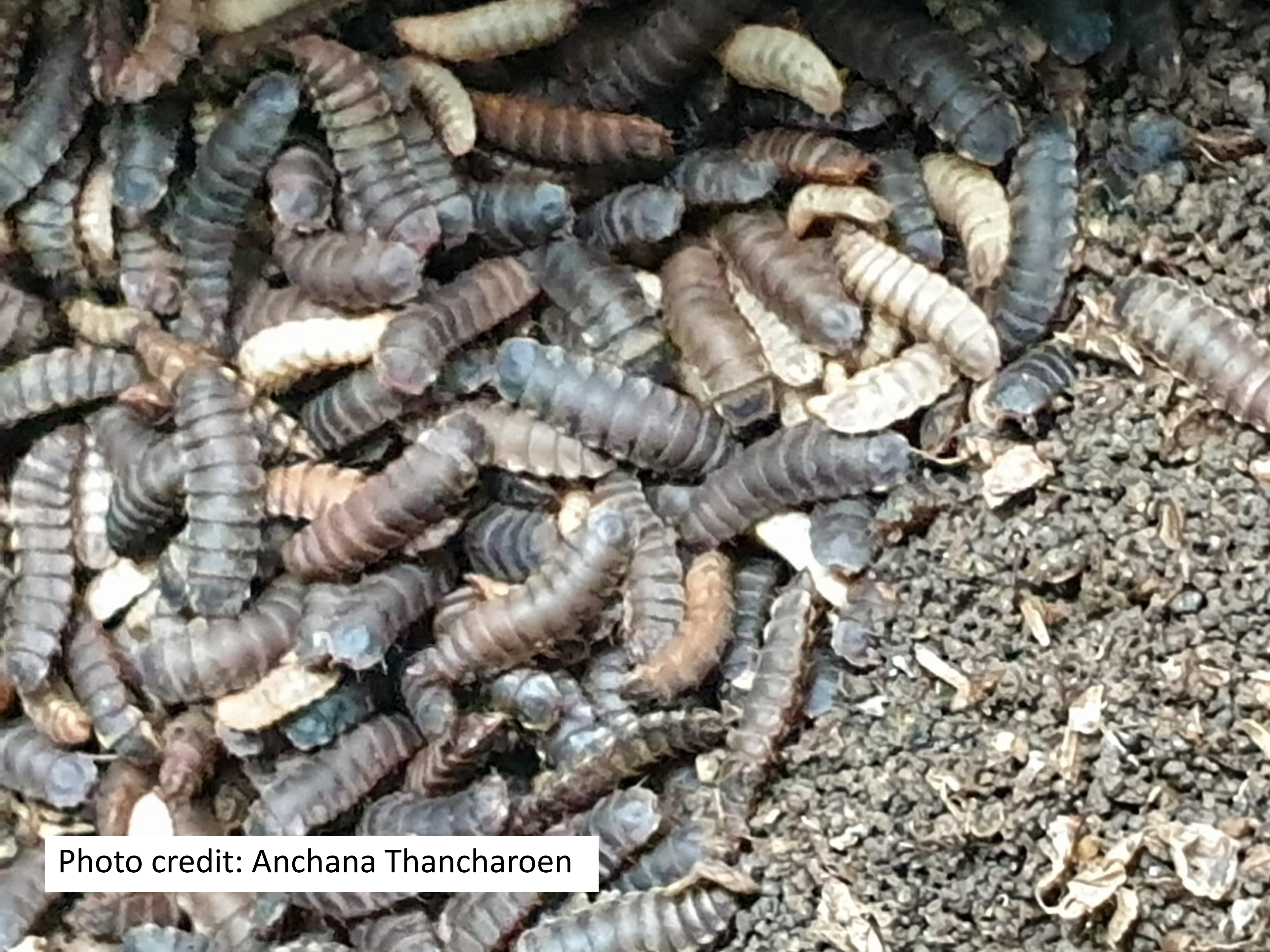

Photo credit: Anchana Thancharoen

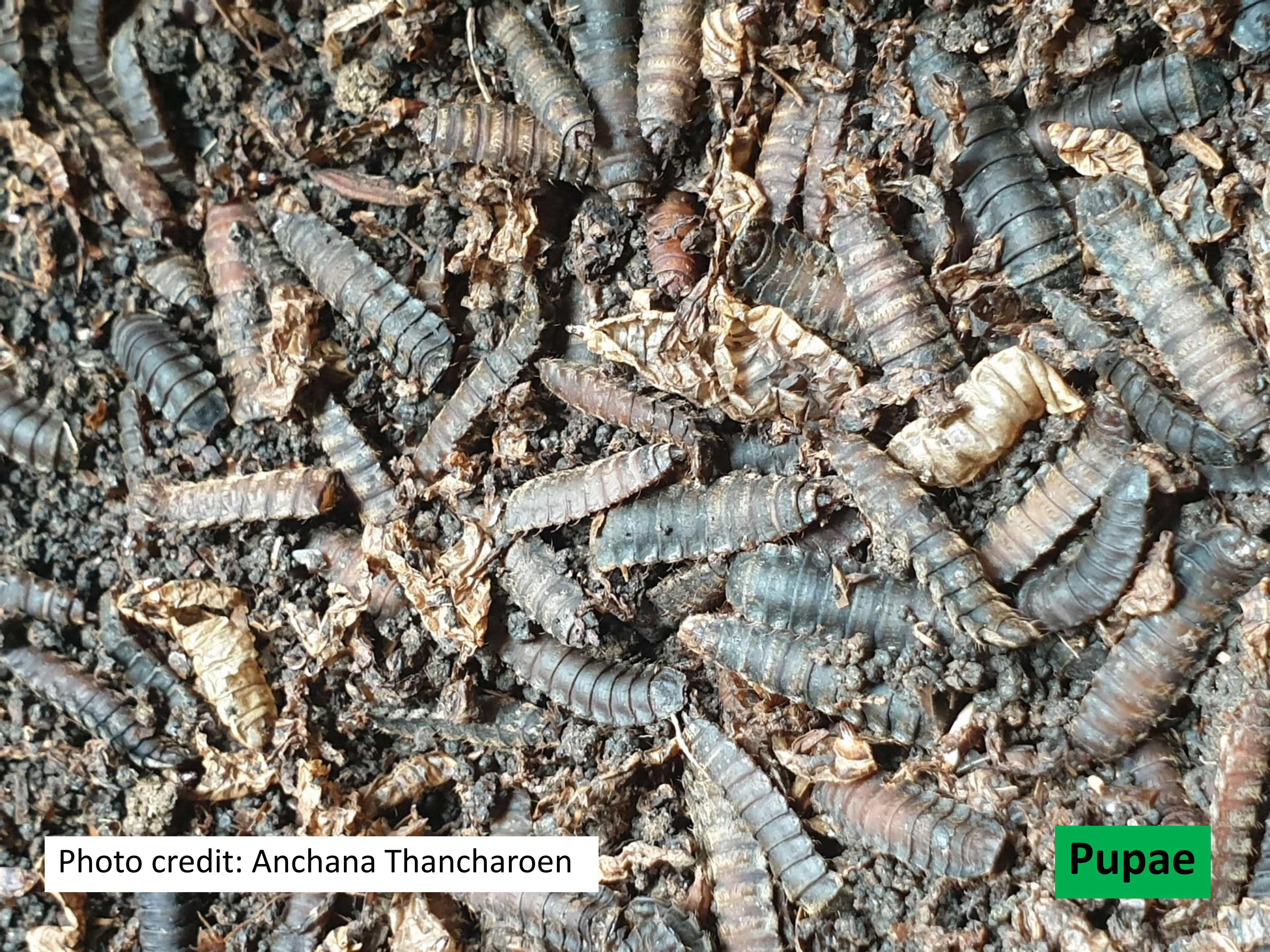
Photo credit: Anchana Thancharoen
Pupae
